# Core–Shell ZnO_2_@Cerium-Based Metal–Organic Framework with Low Turnover, Dual-Catalytic Activity for Biosafe Biofilm Dispersal and Immune Modulation

**DOI:** 10.1021/acsami.5c08974

**Published:** 2025-05-21

**Authors:** Renfei Wu, Tianjin Ge, Tianrong Yu, Qiaolan Shi, Rui Shi, Yijin Ren, Henk J. Busscher, Jian Liu, Henny C. van der Mei

**Affiliations:** † Institute of Functional Nano and Soft Materials, Jiangsu Key Laboratory for Carbon-Based Functional Materials and Devices, 12582 Soochow University, Suzhou, Jiangsu 215123, China; ‡ Department of Biomaterials and Biomedical Technology, University of Groningen and University Medical Center Groningen, Antonius Deusinglaan 1, 9713 AV Groningen, The Netherlands; § Department of Orthodontics, University of Groningen and University Medical Center of Groningen, Hanzeplein 1, 9700 RB Groningen, The Netherlands

**Keywords:** biofilm, extracellular DNA, immune modulation, metal−organic framework, sepsis, turnover frequency, turnover number

## Abstract

The era of relying on antibiotics for curing bacterial infections is rapidly approaching an end, necessitating development of non-antibiotic-based infection-control strategies. Dispersal of infectious biofilms is a potential strategy but yields dispersed bacteria in blood that may cause sepsis. We report a bromide-loaded, core–shell ZnO_2_-nanoparticle/Ce-based metal–organic framework (ZnO_2_@CeMOF/Br) of which the ZnO_2_ core degrades at pH ≤ 6.5, leaving the MOF’s Ce node intact. ZnO_2_-core degradation initially generates a nonradical, relatively stable, low-oxidative hydrogen peroxide that can cleave matrix DNA causing dispersal of *Staphylococcus aureus* biofilms and reacts with bromide ions to form transient hypobromous acid. Hypobromous acid modulates macrophage polarization toward an M1-like phenotype to clear dispersed bacteria from blood. Subsequently the Ce^3+^/Ce^4+^ redox couple forming the Ce node acts as an electron shuttle upon oxidation/reduction to faciltate two catalytic reactions, maintaining hydrolysis of phosphodiester bonds and associated cleavage of matrix DNA as well as modulation of macrophage polarization. Neither growth of tissue cells or macrophages nor hemolysis are negatively affected by exposure to ZnO_2_@CeMOF/Br nanocatalysts at a ZnO_2_ nanoparticle over CeMOFs weight ratio ≤ 1.2, up until CeMOF concentrations less than at least 180 μg/mL. Under biosafe, low-turnover catalytic conditions, irrigation of infected wounds in diabetic mice with ZnO_2_@CeMOF/Br nanocatalysts (90 μg/mL) results in 100% survival, fast recovery of healthy body temperature and weight, lower numbers of CFUs in blood and wound and organ tissues, and macrophage polarization toward an M1-like phenotype, demonstrating potential of ZnO_2_@CeMOF/Br nanocatalysts for non-antibiotic-based infection control.

## Graphical abstract

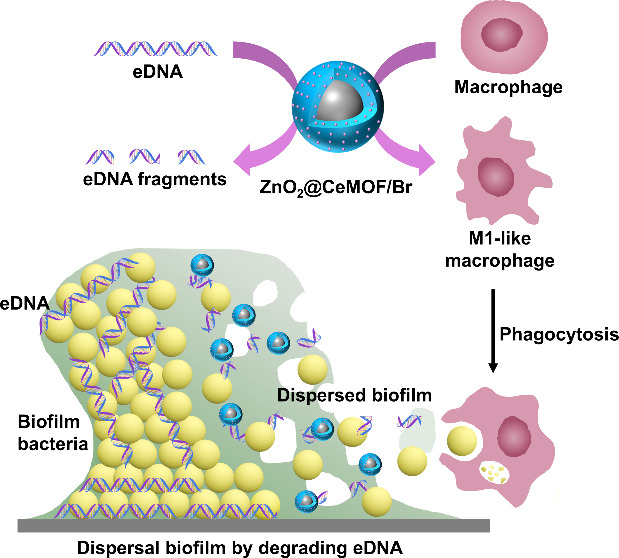

## 1 Introduction

Since the discovery of antibiotics, medicine has relied heavily on the use of antibiotics to cure bacterial infections.^1,2^ Whereas antibiotic treatment can be life-saving, non-antibiotic-based treatment of infection has several advantages, including the maintenance of a balanced healthy microbiome, avoidance of antibiotic side effects like nausea, diarrhea, allergic reactions, and other, more severe side effects, and last, but not least, natural strengthening of the host immunity.^3^ Moreover, the era of being able to rely on antibiotics for curing bacterial infections is rapidly coming to an end, and alternative, non-antibiotic-based infection-control strategies are needed. Dispersal of infectious biofilms is one such strategy but heavily relies on the presumption that the immune system is able to clear the sudden high concentration of dispersed bacteria from the blood circulation. Unfortunately, however, relying on the host immune system has an unpredictable efficacy and may take more time than is available in critical cases of bacterial infection. Also, the host immune system has lower efficacy in elderly patients and patients with an underlying disease such as diabetes or an elsewise compromised immune system.^4–6^ Strengthening of the host immune system can be done by lifestyle-related measures (healthy diet, regular exercise, adequate sleep, stress management) or vaccination, but here too efficacy is unpredictable and often insufficient. Macrophages play a vital role in the clearance of infection by the host immune system.^7,8^ Macrophages present themselves in a wide spectrum of different phenotypes to perform a myriad of functions to maintain tissue homeostasis, phagocytosis, modulation of inflammation, and clearance of infection.^9,10^ Macrophages can switch between a more pro-inflammatory, M1-like phenotype and an anti-inflammatory, more M2-like phenotype. Strengthening of the immune system for the control of bacterial infections accordingly involves modulating macrophage polarization toward a more M1-like phenotype.^7,8^ Recently, nanotechnology has provided immune-modulating nanoparticle-based methodologies to enhance the efficacy of the host immune system toward bacterial infection in which oxidative stress plays a crucial role.^11,12^

Reactive oxygen species (ROS) constitute a class of naturally occurring compounds, comprising nonradical, relatively stable, low-oxidative hydrogen peroxide and free radical, highly transient, high-oxidative hydroxyl radicals and singlet oxygen.^13^ Particularly free-radical ROS have been found to modulate macrophage polarization toward an M1-like phenotype.^14–16^ Hypobromous acid is a transient halogen compound also possessing a high-oxidative power that naturally occurs in blood at low concentrations (<10 μM).^17^ Hypobromous acid has been found in immune cells during response to infections.^18,19^ Although this suggests the role of hypobromous acid in macrophage polarization, its detailed physiological role is unknown.

The transient nature (several microseconds) of high-oxidative free-radical ROS and hypobromous acid impedes harmful spreading to healthy host tissues. However, their short lifetime also makes application of ROS and hypobromous acid generated by novel antimicrobial compounds, such as chitosan and polydopamine-modified poly­(vinyl alcohol) hydrogel coatings,^20^ metastable CuFe_5_O_8_ nanocubes,^15^ or zirconium- or cerium-based metal–organic frameworks (MOFs),^16^ highly difficult. Due to their transient nature, generation must occur close to a target site. Targeting of macrophages in blood by transient compounds for modulatory purposes is relatively easy compared with targeting of bacterial pathogens protected in a biofilm matrix impeding antimicrobial compound penetration. Therewith, generation of oxidizing compounds close to target bacteria inside a biofilm is hard to achieve, making bacterial killing within the compound’s lifetime difficult.

The Ce node in a Ce-based MOF forms a Ce^3+^/Ce^4+^ redox couple that can be oxidized/reduced from Ce^3+^ to Ce^4+^ and vice versa as a characteristic of lanthanide redox couples.^21,22^ In this article, we make use of this unique catalytic property of CeMOFs arising from the multiple oxidation states of Ce as an alternative for antibiotic-based bacterial infection control. Incorporation of ZnO_2_ and bromide ions within a CeMOF (Figure 1A) will initially yield generation of nonradical, low-oxidative hydrogen peroxide from ZnO_2_ nanoparticles degrading in an acidic biofilm environment, causing (I) hydrolysis of phosphodiester bonds in DNA, as one of the major glue components of bacterial biofilm matrices, and (II) the conversion of nonradical hydrogen peroxide and bromide ions into transient, high-oxidative hypobromous acid (Figure 1B). These two initial reactions are hypothesized to simultaneously disperse biofilm bacteria by cleaving biofilm matrix DNA and modulate macrophage polarization toward a more M1-like phenotype (Figure 1C) in order to prepare the host immune system for a high concentration of dispersed bacterial pathogens in the blood circulation. As an important feature of these initial reactions, both the generation of nonradical hydrogen peroxide and hypobromous acid are controlled by the amount of ZnO_2_ nanoparticles inside the CeMOF, preventing the release of ZnO_2_ in concentrations harmful to healthy tissues. The Ce node in the CeMOF is demonstrated to stay intact in an acidic environment using X-ray photoelectron spectroscopy after ZnO_2_ nanoparticle degradation as a catalytic center yielding repetitive hydrolysis of phosphodiester bonds to cleave matrix DNA and generation of hypobromous acid for the modulation of macrophage polarization (Figure 1D). Reaction equations are proposed for both catalytic processes and turnover numbers and frequencies calculated.

**Figure 1:**
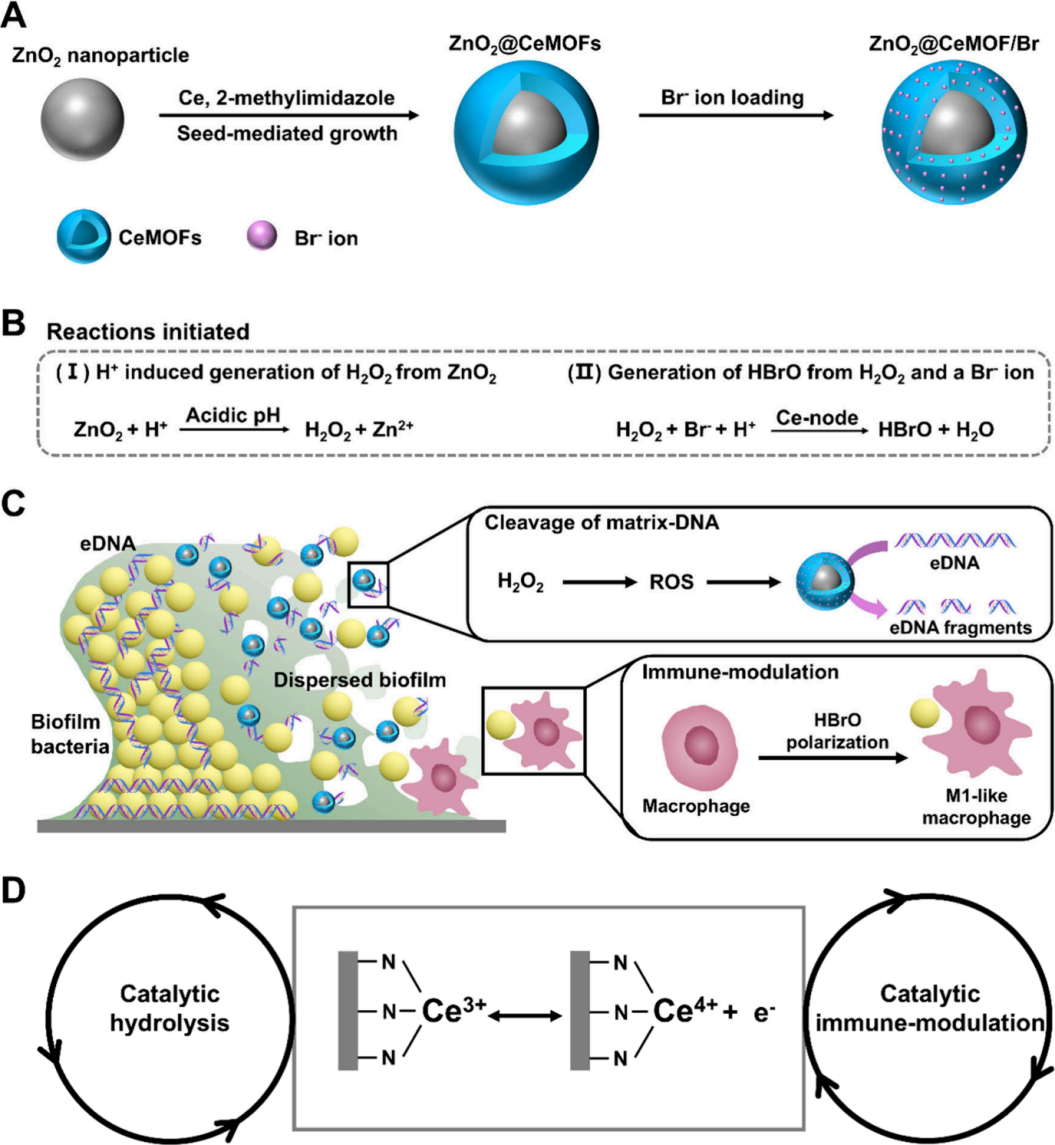
Synthesis, reactions initiated by core–shell ZnO_2_@CeMOF/Br nanocatalysts, and the role of the Ce^3+/^Ce^4+^ redox couple in the hypothesized, dual-working mechanism of core–shell ZnO_2_@CeMOF/Br nanocatalysts, yielding cleavage of matrix DNA and immune modulation. (A) Synthesis of core–shell ZnO_2_@CeMOFs by seed-mediated growth, utilizing 2-methylimidazole as an organic linker, including loading with Br^–^ ions in the pores of Ce-based MOFs. (B) Reactions initiated by ZnO_2_@CeMOF/Br nanocatalysts in an acidic environment: (I) H^+^-induced generation of nonradical hydrogen peroxide (H_2_O_2_) from ZnO_2_ and (II) conversion of bromide ions (Br^–^) and nonradical hydrogen peroxide into transient hypobromous acid (HBrO). (C) Hypothesized dual-working mechanism of core–shell ZnO_2_@CeMOF/Br nanocatalysts based on the initial reactions provoked, yielding dispersal of biofilm bacteria and modulation of macrophage polarization toward a more M1-like phenotype. (D) Intact Ce node in CeMOFs after degradation of ZnO_2_ nanoparticles providing a redox couple hypothesized to facilitate catalytic hydrolysis of phosphodiester bonds in DNA and generation of hypobromous acid.

## 2 Results

### 2.1 ZnO_2_ Nanoparticles Encapsulated in a CeMOF

ZnO_2_ nanoparticles with a diameter of 51 nm (Figure 2A) were utilized as a core template for seed-mediated growth of core–shell ZnO_2_@CeMOFs, as outlined in Figure 1A. CeMOFs, with a mean diameter of 70 nm (Figure 2B) were constructed on the surface of ZnO_2_ nanoparticles through coordination interaction between imidazole groups of the CeMOFs and Zn^2+^ ions (Figure 2C) at a ZnO_2_ over CeMOF weight ratio of 1.2 to ensure minimal cytotoxic effects (see Section 2.2). Interaction yielded highly monodisperse nanoparticles with a diameter (79 nm; Figure 2D) larger than that of ZnO_2_ nanoparticles or CeMOFs. Elementary mapping of the composition of the core–shell CeMOFs, synthesized using high-angle annular dark-field scanning TEM (HAADF-STEM), showed the core to be composed of Zn, surrounded by a Ce shell (Figure 2E). In line, the ζ potential of ZnO_2_ nanoparticles amounted to +20.0 mV and increased to +34.5 mV upon encapsulation in a CeMOF, similar to that observed for CeMOFs (+33.1 mV; Figure 2F), confirming successful construction of a core–shell structure.

**Figure 2:**
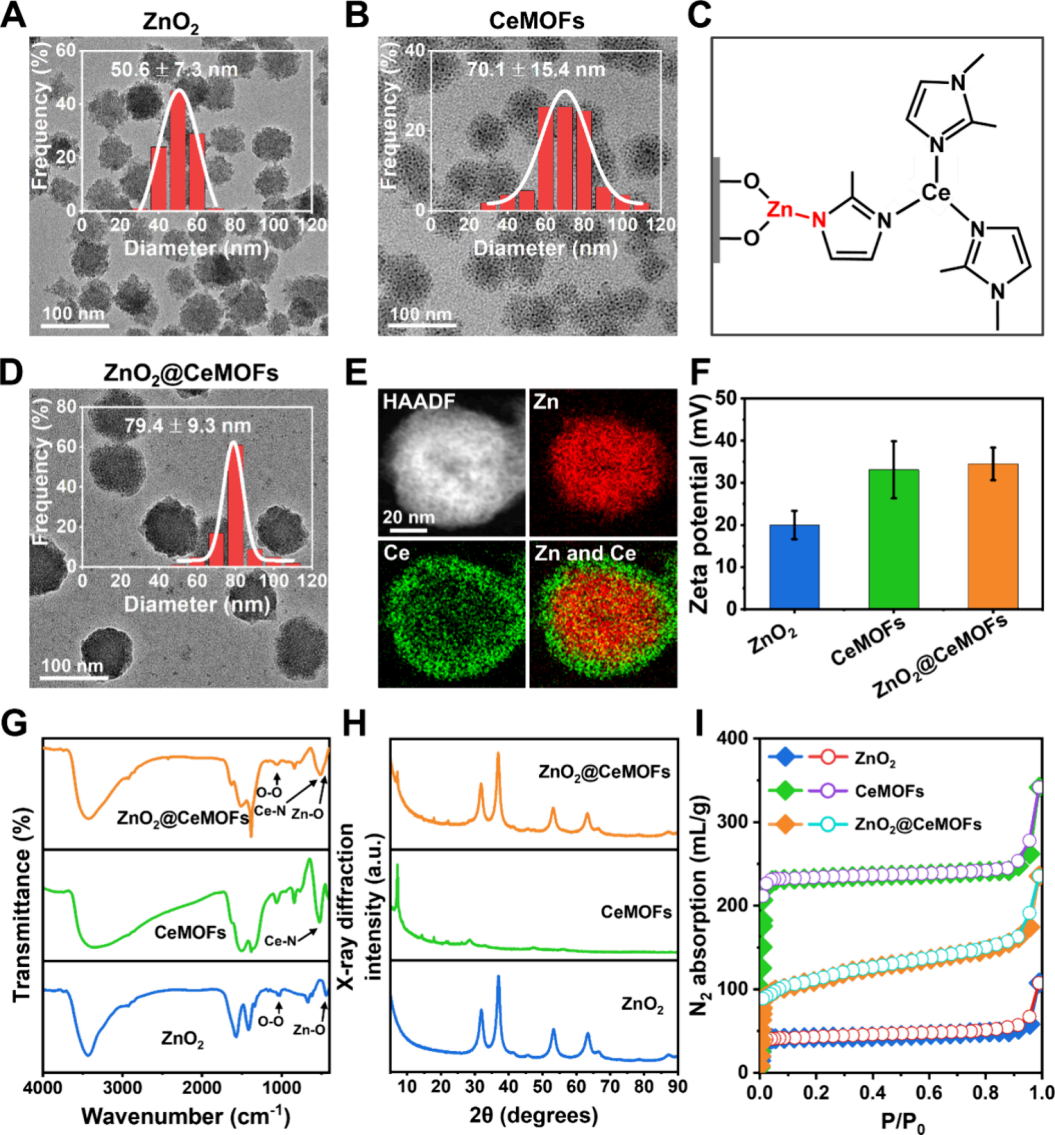
Synthesis and characterization of core–shell ZnO_2_@CeMOFs in the absence of bromide loading. ZnO_2_@CeMOFs have been prepared at a weight ratio of ZnO_2_ and CeMOF of 1.2 (see also Section 2.2). (A) TEM micrograph of ZnO_2_ nanoparticles, with diameter distribution obtained by measuring the diameters of 100 nanoparticles (bin size, 10 nm). (B) Same as panel A, now for CeMOFs. (C) Coordination interaction between imidazole groups and Zn^2+^ ions, facilitating seed-mediated growth of CeMOFs, encapsulating ZnO_2_ nanoparticles. (D) TEM micrograph of ZnO_2_@CeMOFs (for details, see Figure 2A). (E) High-angle annular dark-field (HAADF), scanning TEM micrograph of single ZnO_2_@CeMOFs and corresponding energy-dispersive X-ray mapping of Zn and Ce in core–shell ZnO_2_@CeMOFs, clearly distinguishing the Zn-rich core and the Ce-rich shell. (F) ζ potentials of ZnO_2_, CeMOFs and ZnO_2_@CeMOFs in 10 mM potassium phosphate buffer (pH 7.4). Data represent means over triplicate experiments with error bars indicating standard deviations. (G) FT-IR spectra of ZnO_2_ nanoparticles, CeMOFs and ZnO_2_@CeMOFs, with arrows indicating characteristic absorption bands. (H) X-ray diffraction patterns of ZnO_2_ nanoparticles, CeMOFs and core–shell ZnO_2_@CeMOFs. (I) Nitrogen (N_2_) adsorption–desorption isotherms measured at 77 K of ZnO_2_ nanoparticles, CeMOFs and core–shell ZnO_2_@CeMOFs. The solid and open symbols (mainly overlapping) represent absorption and desorption, respectively.

FT-IR absorption bands characteristic of ZnO_2_ (1040 cm^–1^ due to O–O stretching, 436 cm^–1^ due to Zn–O stretching) and CeMOFs (524 cm^–1^ due to Ce–N stretching), could both be found in the spectrum of ZnO_2_@CeMOFs (Figure 2G). X-ray diffraction indicated that the crystallinity of ZnO_2_ nanoparticles (32, 37, and 53°) and CeMOFs (7, 14, and 18°) were preserved upon seed-mediated growth of the core–shell structure (Figure 2H). CeMOFs possessed a very high BET surface area of 683 m^2^/g as derived from N_2_ adsorption–desorption isotherms (Figure 2I), that was reduced in ZnO_2_@CeMOFs to 317 m^2^/g due to the presence of ZnO_2_ nanoparticles. Note that the adsorption and desorption isotherms of CeMOFs overlapped, implying that CeMOFs possess well-ordered, channels.

### 2.2 In Vitro Cytotoxicity and Hemolysis of Core–Shell ZnO_2_@CeMOFs Controlled by the ZnO_2_ Nanoparticle over CeMOFs Weight Ratio and Not Affected by Bromide-Ion Loading

The initial reactions and catalytic effects aimed for (see Figure 1) required a balanced synthesis of ZnO_2_@CeMOFs with a ratio of ZnO_2_ nanoparticles over CeMOFs possessing minimal cytotoxicity, yet allowing ZnO_2_ degradation and generation of H_2_O_2_. Growth of NIH 3T3 fibroblasts, human umbilical vein endothelial cells (HUVECs) and J774A.1 macrophages in the presence of ZnO_2_ nanoparticles or CeMOFs at different concentrations (Figure 3A) demonstrated that CeMOFs had no negative effect on cell viability up to MOF concentrations of at least 180 μg/mL. However, cell viability decreased with increasing concentrations of ZnO_2_ nanoparticles. This suggests that encapsulation in CeMOFs may mitigate the cytotoxicity of the ZnO_2_ nanoparticles encapsulated. This suggestion was confirmed in Figure 3B, demonstrating the absence of cytotoxic effects of ZnO_2_@CeMOFs prepared at a ZnO_2_ nanoparticle over CeMOFs weight ratio of ≤ 1.2, regardless of CeMOF concentration up until at least 180 μg/mL. For these ratios and concentrations, hemolysis of mouse red blood cells exposed to ZnO_2_, CeMOFs, and ZnO_2_@CeMOFs was <5% (Figure 3C), indicating negligible hemolysis. Using inductively coupled plasma mass spectrometry (ICP-MS), the wt % bromide loaded into ZnO_2_@CeMOFs was found to be 40 ± 15%. Bromide-ion loading of ZnO_2_@CeMOFs did not significantly affect the cytotoxicity or hemolytic activity of the MOFs (Supporting Information Figure S1). Consequently, all further experiments in this article were done at a ZnO_2_ nanoparticle over CeMOF ratio of 1.2 at a CeMOF concentration of ≤180 μg/mL.

**Figure 3:**
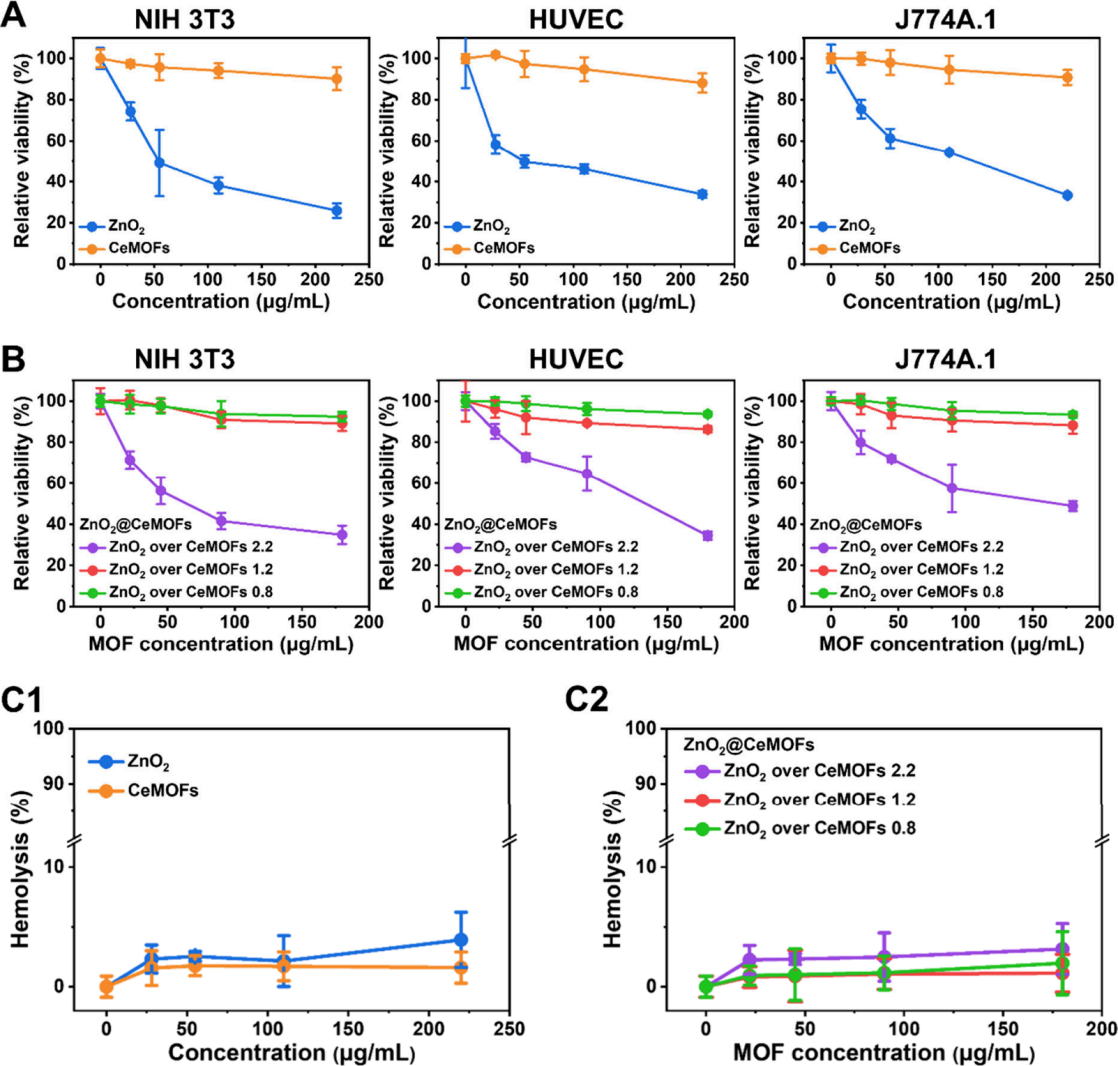
Cytotoxicity of ZnO_2_ nanoparticles, CeMOFs and ZnO_2_@CeMOFs synthesized at different ratios of ZnO_2_ over CeMOFs toward NIH 3T3 fibroblasts, human umbilical vein endothelial cells (HUVECs), and J774A.1 macrophages. (A) Relative viability of fibroblasts, HUVECs, and macrophages, grown in the presence of different concentrations of ZnO_2_ nanoparticles or CeMOFs. Viability was expressed with respect to growth medium without ZnO_2_ nanoparticles and CeMOFs, set at 100%. (B) Same as panel A, now for growth in the presence of ZnO_2_@CeMOFs. Data are presented in CeMOFs equivalent concentrations. (C) Hemolysis of mouse red blood cells after 3 h of exposure to different concentrations of ZnO_2_ nanoparticles, CeMOFs and ZnO_2_@CeMOFs. (C1) Data for ZnO_2_ nanoparticles and CeMOFs. (C2) Data for ZnO_2_@CeMOFs. Hemolysis was derived from UV absorbance at 540 nm, setting hemoglobin absorption of cells exposed to ultrapure water as 100%, and that exposed to PBS as 0%. Data represent means over five experiments with separately prepared cell cultures, and error bars indicate standard deviations.

### 2.3 Zn and Bromide Ions Released from ZnO_2_@CeMOF/Br Nanocatalysts in an Acidic Environment, Generating Nonradical Hydrogen Peroxide and Transient Hypobromous Acid while Leaving the Ce node Intact

ZnO_2_@CeMOFs decomposed in an acidic environment to release Zn. Zn release increased gradually over time and with a decrease in pH (Figure 4A). Release of Ce from the CeMOFs could not be observed (Figure 4B), suggesting that the CeMOFs remain intact, as confirmed by TEM (Figure 4C). Thus, only the ZnO_2_ core is responsive to acid-induced degradation, while the CeMOF shell is stable with an intact Ce node. In line with our hypotheses as outlined in Figure 1B, nonradical hydrogen peroxide generation in the presence of ZnO_2_@CeMOFs, gradually increased over time and with decreasing pH (Figure 4D). Generation of transient hypobromous acid by ZnO_2_@CeMOF/Br nanocatalysts (see also Figure 1B) occurred only upon bromide-ions loading of ZnO_2_@CeMOFs (Figure 4E), also gradually increasing over time and with decreasing pH. Concurrent with the acid-induced degradation of the ZnO_2_ core that was accompanied by the consumption of bromide ions and generation of hypobromous acid, the negative zeta potential of ZnO_2_@CeMOF/Br nanocatalysts (−10.4 mV) became positive (+13.7 mV: see also Figure 4F) due to the positive charge of the Ce node left intact after degradation of the ZnO_2_ nanoparticles. X-ray photoelectron spectroscopy indicated the existence of Ce in its 3+ and 4+ oxidation states before and after catalysis (Figure S2) and therewith confirms that the Ce^3+/^Ce^4+^ redox couple in the CeMOF stays intact during catalysis.

**Figure 4:**
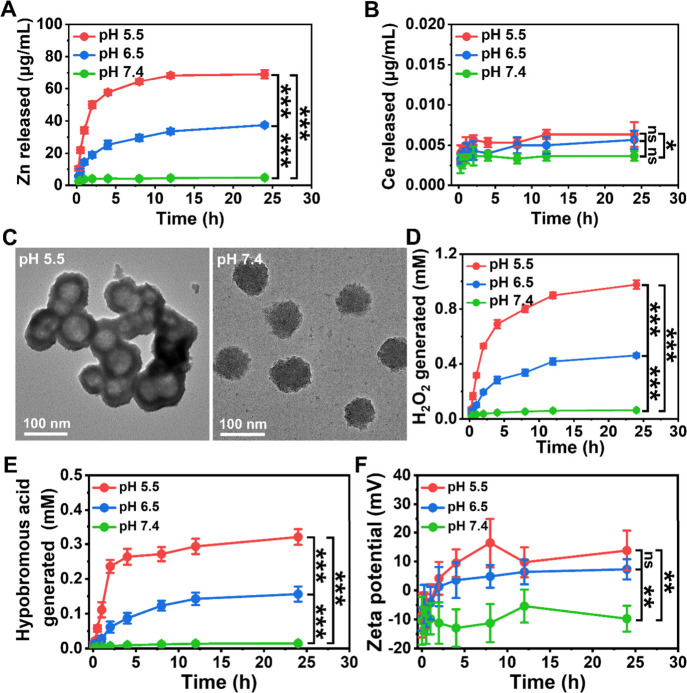
Acid-responsive core–shell ZnO_2_@CeMOFs and bromide-ion loading at a CeMOF concentration of 90 μg/mL. (A) Release of Zn from ZnO_2_@CeMOF/Br nanocatalysts as a function of exposure time in 10 mM potassium phosphate buffers with different pH. (B) Same as panel A, now for the release of Ce. (C) TEM micrographs of ZnO_2_@CeMOF/Br nanocatalysts after 24 h exposure to phosphate buffer. (D) Total amount of nonradical hydrogen peroxide (H_2_O_2_) generated from ZnO_2_@CeMOF nanocatalysts as a function of time during exposure to phosphate buffer with different pH, measured using a Hydrogen Peroxide Assay Kit (see Figure S3). (E) Same as panel D, now for the generation of transient hypobromous acid by ZnO_2_@CeMOF/Br nanocatalysts, measured from the bromination of phenol red to bromophenol blue (see Figure S4). (F) ζ potentials of bromide-loaded ZnO_2_@CeMOF/Br nanocatalysts as a function of exposure time to 10 mM potassium phosphate buffers with different pH. Data represent means over triplicate experiments, and error bars indicate standard deviations. Statistically significant differences between pairs of data are indicated by the vertical spanning bars (*p < 0.05; **p < 0.01; ***p < 0.001; two-tailed Student’s *t* test). ns indicates no significance.

### 2.4 Phosphodiester Bonds Hydrolyzed by Bromide-Loaded ZnO_2_@CeMOF/Br Nanocatalysts Yielding Biofilm Dispersal In Vitro

In order to evaluate the ability of ZnO_2_@CeMOF/Br to hydrolyze phosphodiester bonds as occurring in extracellular DNA of biofilm matrices, bis­(4-nitrophenyl)­phosphate (BNPP) was utilized. BNPP possesses similar phosphodiester bonds as DNA (see Figure S5A) but is easier to obtain. Hydrolysis of phosphodiester bonds in BNPP leads to the formation of nitrophenolate with a characteristic UV–vis absorbance at 400 nm (Figure S5B). ZnO_2_ had no ability to hydrolyze phosphodiester bonds, but both CeMOFs with and without a ZnO_2_ core as well as ZnO_2_@CeMOF/Br nanocatalysts demonstrated similarly high abilities to hydrolyze phosphodiester bonds (Figure 5A). Hydrolysis of phosphodiester bonds by ZnO_2_@CeMOFs with or without bromide-ion loading extended to cleavage of extracellular matrix DNA to cause dispersal of 24 h old, *Staphylococcus aureus* (*S. aureus*) Xen36 biofilms (see Figure 5B and Figures S6 and S7 for CLSM images). Dispersal is due to a reduced glueing power of the biofilm matrix, i.e., hydrolysis of phosphodiester bonds in extracellular DNA, and left a more open biofilm structure. Concurrent with the development of a more open biofilm structure, a concentration dependent decrease in biofilm thickness was observed (Figure 5C). Combining the biofilm thickness and the number of CFUs retrieved from remaining biofilm per unit area allowed the expression of the openness of the biofilm structure as a volumetric CFU density in a biofilm as a quantitative measure of dispersal. Thus, obtained volumetric CFU densities of biofilms exposed to ZnO_2_@CeMOFs with bromide ion loading decreased with MOF concentration, leveling off at 90 μg/mL (Figure 5D), regardless of bromide loading. Hence 90 μg/mL was taken as a MOF concentration in further experiments, well within the limit of adverse cytotoxic and hemolytic effects determined above.

**Figure 5:**
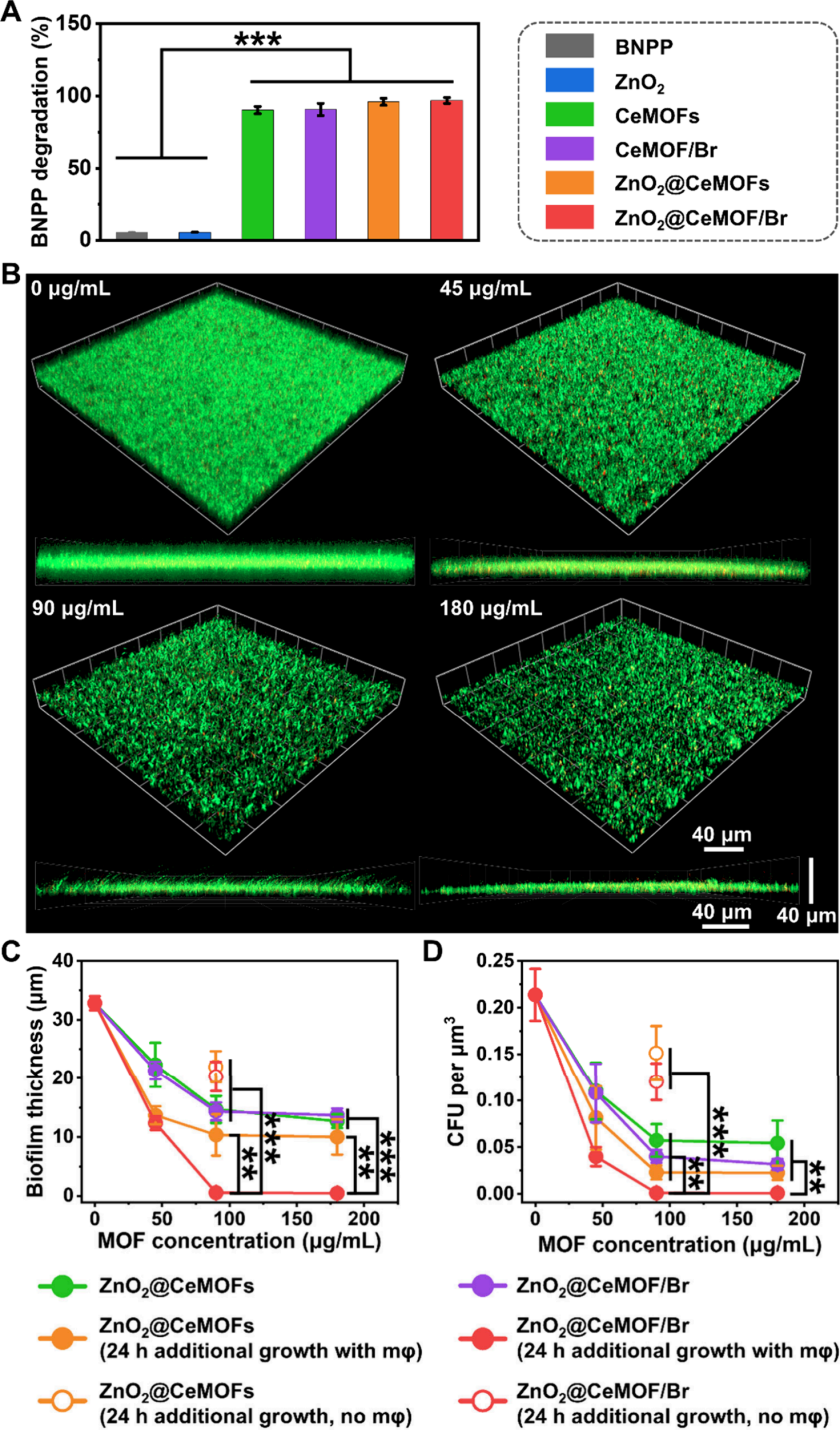
Hydrolysis of phosphodiester bonds in BNPP and dispersal of 24 h of *S. aureus* Xen36 biofilms by ZnO_2_, CeMOFs, and ZnO_2_@CeMOFs, with and without bromide-ion loading in vitro and in the absence and presence of macrophages (mφ). (A) Hydrolysis of phosphodiester bonds in BNPP upon 24 h exposure to ZnO_2_ nanoparticles (concentration, 110 μg/mL), CeMOFs and ZnO_2_@CeMOFs with and without bromide-ion loading (CeMOF concentration, 90 μg/mL) suspended in potassium phosphate buffer (10 mM, pH 5.5). Hydrolysis was calculated from the absorbance at 400 nm due to nitrophenolate arising due to hydrolysis of phosphodiester bonds (Figure S5C) and expressed relative to the absorbance of nitrophenolate arising from hydrolysis in 0.4 mM BNPP. (B) CLSM overlay and cross-sectional images of 24 h old biofilms exposed for 24 h to different concentrations of ZnO_2_@CeMOF/Br nanocatalysts. (C) Biofilm thickness as a function of the concentration of ZnO_2_@CeMOFs (see Figure S6A for CLSM images) and ZnO_2_@CeMOF/Br nanocatalysts (see panel B for images). Data in the presence of macrophages were derived by an additional 24 h growth in the presence of macrophages, following initial exposure to ZnO_2_@CeMOFs (see Figure S6B for CLSM images) and ZnO_2_@CeMOF/Br nanocatalysts (see Figure S7 for images). Note, open symbols refer to data obtained by 24 h of additional growth in full medium in the absence of macrophages, following initial exposure to ZnO_2_@CeMOFs and ZnO_2_@CeMOF/Br nanocatalysts. (D) Same as panel C, now for the number of CFUs per unit volume in the remaining biofilm. Volumetric bacterial densities in biofilms were calculated as the number of CFUs cultured from a specific biofilm volume, divided by the biofilm volume derived from CLSM images as in panel B. Data represent means over triplicate experiments with separately cultured bacteria and error bars indicating standard deviations. Statistically significant differences between pairs of data are indicated by the spanning bars (**p < 0.01;***p < 0.001; two-tailed Student’s *t* test).

### 2.5 Macrophages Modulated toward an M1-like Phenotype by Bromide-Ion-Loaded ZnO_2_@CeMOF/Br Nanocatalysts In Vitro

The impact of ZnO_2_@CeMOF/Br nanocatalysts on macrophage polarization was assessed by measuring the secretion of TNF-α and IL-6, indicative of the M1 phenotype, using different enzyme-linked immunosorbent assays (see Figure S8). Macrophages exposed to phosphate-buffered saline (PBS), ZnO_2_ nanoparticles, CeMOFs, CeMOF/Br, or ZnO_2_@CeMOFs displayed relatively low levels of TNF-α (Figure 6A) and IL-6 (Figure 6B) secretion. In contrast, only exposure to ZnO_2_@CeMOF/Br nanocatalysts resulted in higher secretions of TNF-α and IL-6, indicating that ZnO_2_@CeMOF/Br nanocatalysts modulated macrophage polarization toward the M1 “fighting” phenotype. This polarization pattern induced by ZnO_2_@CeMOF/Br nanocatalysts, as evidenced by the cytokine expressions, resembled the effect achieved by exposure to lipopolysaccharides or exogenous hypobromous acid (see also Figure 6) and demonstrates that ZnO_2_@CeMOF/Br nanocatalysts modulate macrophages to polarize toward the M1 phenotype through generation of hypobromous acid.

**Figure 6:**
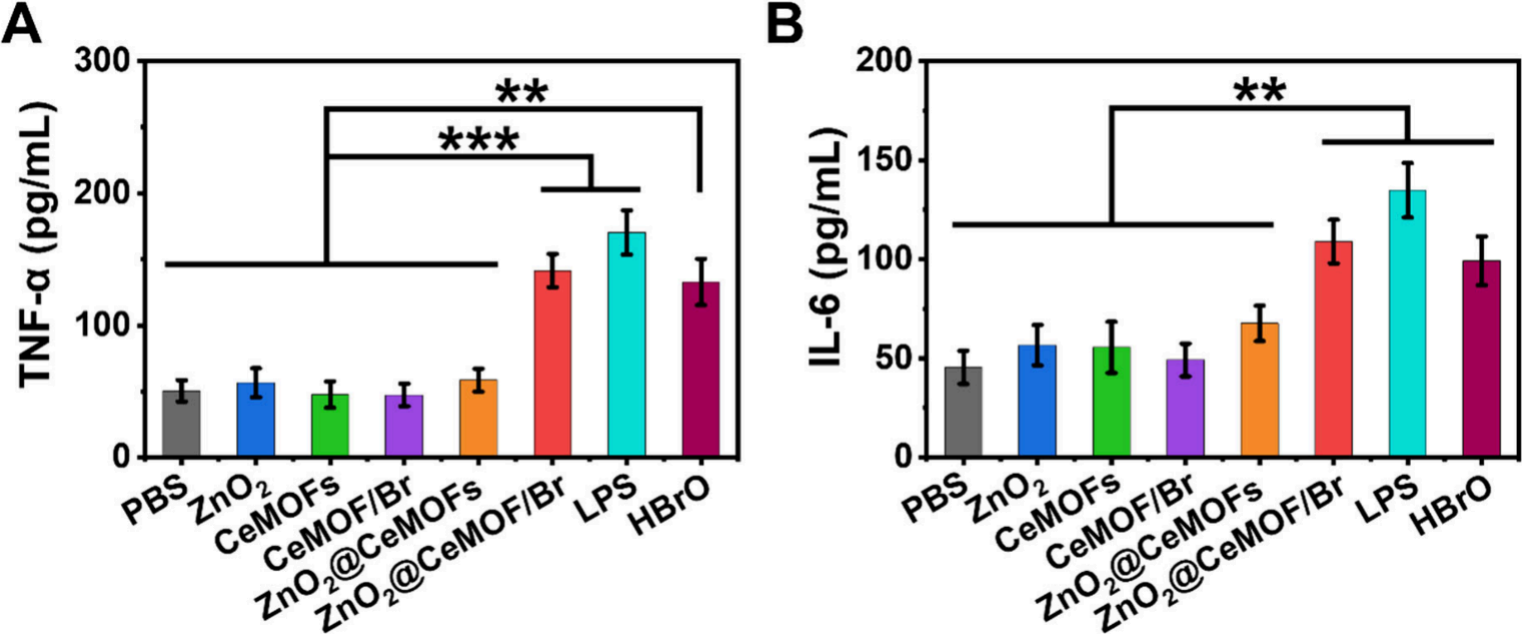
Modulation of macrophage polarization toward an M1-like phenotype upon 24 h exposure to ZnO_2_@CeMOF/Br nanocatalysts. Macrophages in Dulbecco’s modified Eagle medium high glucose (DMEM-HG) were exposed to ZnO_2_ nanoparticles (ZnO_2_ concentration, 110 μg/mL), CeMOFs, and ZnO_2_@CeMOFs with and without bromide-ion loading (MOF concentration, 90 μg/mL) suspended in DMEM-HG or to lipopolysaccharides (LPS, 10 μg/mL) and exogenous hypobromous acid (5 μg/mL) in DMEM-HG. Secretions of TNF-α and IL-6 were measured using different enzyme-linked immunosorbent assays (see Figure S8 for calibration curves). (A) TNF-α secretion. (B) IL-6 secretion. Data represent means over triplicate experiments with separately cultured macrophages, and error bars indicate standard deviations. Statistically significant differences between pairs of data are indicated by the horizontal spanning bars (**p < 0.01; ***p < 0.001; two-tailed Student’s *t* test).

In Figure 5C,D it can also be seen that 24 h growth of *S. aureus* Xen36 biofilms to macrophages following 24 h initial exposure to ZnO_2_@CeMOFs or ZnO_2_@CeMOF/Br nanocatalysts further decreased biofilm thickness and volumetric bacterial density upon bromide-ion loading but not in the absence of bromide-ion loading, evidencing more effective bacterial clearance by M1-like polarized macrophages using ZnO_2_@CeMOF/Br nanocatalysts. Note that growth of biofilms remaining after dispersal at a MOF concentration of 90 μg/mL in medium without macrophages demonstrated fast regrowth of biofilms to roughly half the biofilm thickness and density as observed prior to exposure.

### 2.6 ZnO_2_@CeMOF/Br Nanocatalysts Safely Irrigated on Open Wounds In Vivo

For in vivo evaluation of ZnO_2_@CeMOF/Br nanocatalysts, a murine-infected wound model was employed. First, however, the conclusion regarding biosafety drawn on the basis of in vitro experiments (Figure 3 and Figure S1), stating that ZnO_2_@CeMOF/Br nanocatalysts were not cytotoxic nor hemolytic up to MOF concentrations ≤ 180 μg/mL, was confirmed in vivo by subcutaneous injection in diabetic mice without infection. Injection of 100 μL of ZnO_2_@CeMOF/Br (180 μg/mL MOF concentration) or 100 μL of PBS as a control yielded no aberrations in blood chemistry (Figure S9A) or histology of major organ tissues (Figure S9B), confirming biosafety in vivo.

### 2.7 Treatment of Infected Wounds Using ZnO_2_@CeMOF/Br Nanocatalysts in Mice, Yielding Survival without Antibiotic Assistance

Treatment of diabetic mice with an infected wound under biosafe conditions by irrigation of 100 μL of PBS containing 90 μg/mL CeMOF/Br or ZnO_2_@CeMOFs on the wound led to septic symptoms, comprising significant death (Figure 7A), loss of weight (Figure 7B), and drop in body temperature (Figure 7C). Interestingly in the group treated with a PBS control, death occurred only after 42 h and to a lesser extent than observed upon treatment with CeMOF/Br or ZnO_2_@CeMOFs. This attests to the severity of the infection inflicted on the wound that, left without adequate treatment, led to natural dispersal of biofilm bacteria to cause septic symptoms and eventually death. However, upon injection of ZnO_2_@CeMOF/Br nanocatalysts, all mice survived, while recovery of weight loss and normal body temperature occurred significantly faster. Already 24 h after initiating treatment, all five mice in the group treated with ZnO_2_@CeMOF/Br nanocatalysts had significantly lower CFU counts in blood and wound and organ tissues (Figure 7D1) than surviving mice treated with CeMOF/Br or ZnO_2_@CeMOFs. 72 h after initiating treatment with ZnO_2_@CeMOF/Br nanocatalysts, CFU counts had decreased significantly in surviving mice with respect to the CFU counts after 24 h. Interesting again, CFU counts in blood and wound and organ tissues in surviving mice with infected wounds irrigated with PBS were higher after 72 h (Figure 7D2) than after 24 h (Figure 7D1) due to natural dispersal of biofilm bacteria in the absence of any treatment. Reduced blood counts upon treatment with ZnO_2_@CeMOF/Br nanocatalysts were accompanied by higher levels of TNF-α (Figure 7E) and IL-6 (Figure 7F). These results demonstrate that ZnO_2_@CeMOF/Br nanocatalysts can disperse biofilms and modulate macrophages to polarize toward an M1-like phenotype to eradicate dispersed bacteria from the blood circulation, causing survival of all mice thus treated.

**Figure 7:**
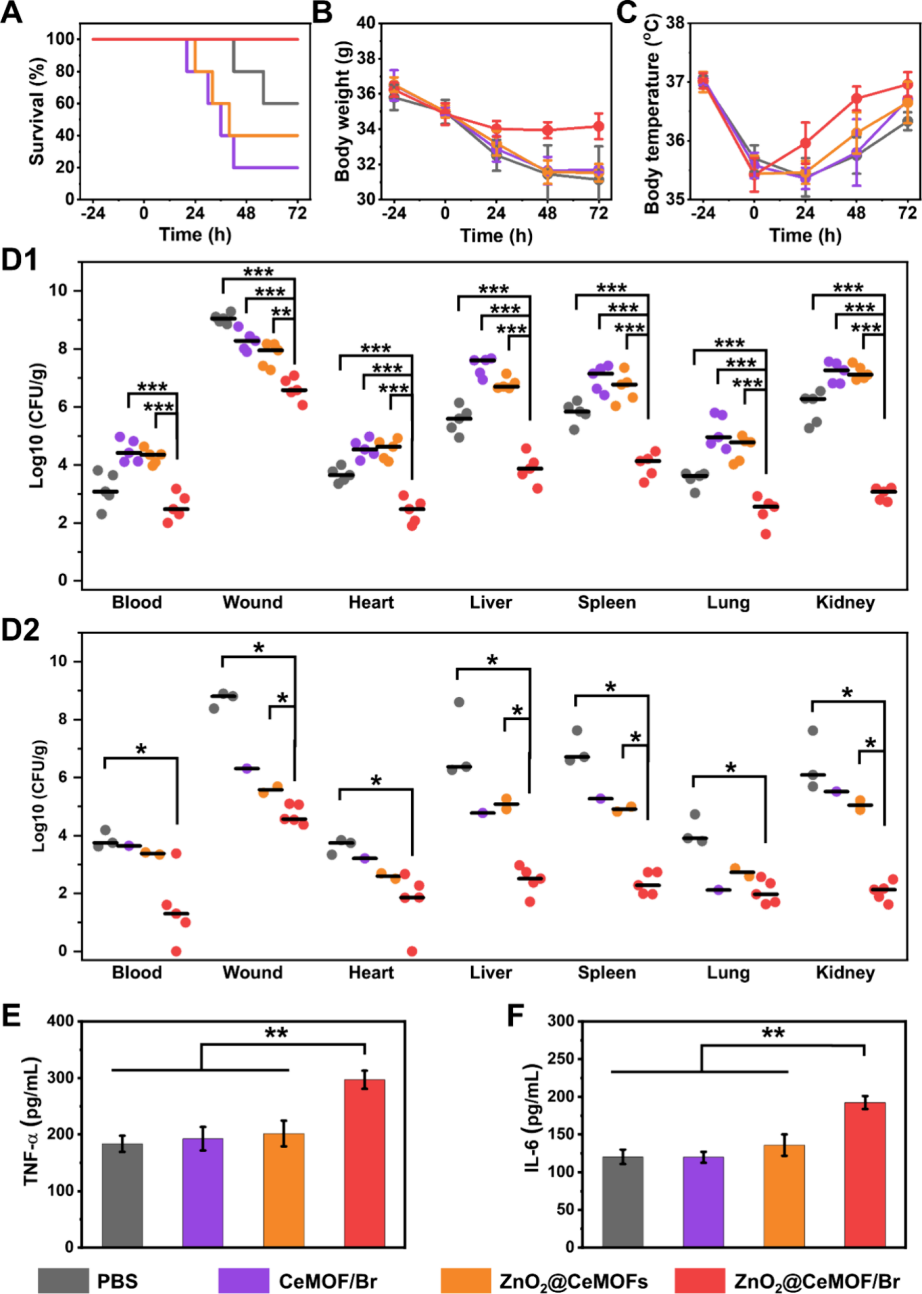
Biosafe treatment of *S. aureus* Xen36 infected wounds using ZnO_2_@CeMOF/Br nanocatalysts in diabetic mice. Infected wounds with an average area of 6 cm^2^ were treated by irrigation with 100 μL of PBS or suspensions with different concentrations of CeMOF/Br, ZnO_2_@CeMOFs, or ZnO_2_@CeMOF/Br nanocatalysts (90 μg/mL MOF concentration). (A) Kaplan–Meijer curve of the survival of mice as a function of time after initiating different treatments. (B) Average body weight of surviving mice as a function of time after initiating treatment. (C) Average body temperature of surviving mice as a function of time after initiating treatment. (D) Number of *S. aureus* Xen36 (CFU/g) in surviving mice (median) retrieved from blood, wound, and other organ tissues of mice sacrificed 24 h (panel D1) and 72 h (panel D2) after initiating treatment (separate group of mice). (E) TNF-α in wounds of surviving mice, sacrificed after 24 h. (F) IL-6 in wounds of surviving mice, sacrificed after 24 h. All data, with the exception of those presented in panel D, represent means over surviving mice in a group of five, with error bars indicating standard deviations. Statistically significant differences between pairs of data are indicated by the spanning bars (*p < 0.05: **p < 0.01; ***p < 0.001; two-tailed Student’s *t* test for panels D1, E, and F and permutation test for panel D2.

## 3 Discussion

In this article, we confirm our hypothesis that incorporation of ZnO_2_ nanoparticles and bromide ions within a CeMOF constitutes a biosafe nanocatalyst with dual-catalytic activity. Core–shell ZnO_2_@CeMOF/Br nanocatalysts initially generate nonradical, low-oxidative hydrogen peroxide from ZnO_2_ in an acidic environment causing dispersal of biofilm bacteria, while conversion of nonradical hydrogen peroxide and bromide ions into transient, high-oxidative hypobromous acid modulates macrophage polarization toward a more M1-like phenotype. Encapsulation of ZnO_2_ nanoparticles in CeMOFs at an appropriate weight ratio less than 1.2 ensures viability of structural, fibroblast cells, vascular, endothelial cells, and immune cells (macrophages) (Figure 3) without adverse effects on blood chemistry and major organ tissues (Figure S9) up to a CeMOF concentration of 180 μg/mL. Therefore, a ZnO_2_@CeMOF/Br concentration of 90 μg/mL was considered biosafe for irrigation of infected wounds in diabetic mice, possessing a compromised immune system in which relatively thick and compact biofilm easily grows.^23^ Irrigation of infected wounds with ZnO_2_@CeMOF/Br nanocatalysts without antibiotic assistance resulted in 100% survival, fast recovery of healthy body temperature and weight, lower numbers of CFUs in blood and wound and organ tissues (Figure 7) and macrophage polarization toward an M1-like phenotype (Figure 6), all in line with the hypothesis forwarded in Figure 1.

The ZnO_2_ nanoparticle core of ZnO_2_@CeMOF/Br nanocatalysts prepared at a ZnO_2_ to CeMOF weight ratio of ≤ 1.2 starts degrading slowly at pH ≤ 6.5, leaving the positively charged Ce node of the CeMOF intact without displaying any in vitro or in vivo cytotoxicity. Generally, both murine and human infected wounds can be alkaline with pH between 7 to 8.^24,25^ Murine infected wounds are more acidic than human wounds,^24,25^ which forms a limitation of the *in vivo* work carried out in mice, pertaining not only to the current study but also to many other articles on infected wounds in mice.^26–30^ However, as infected wounds begin to heal following debridement and effective wound care, pH gradually becomes more neutral or slightly acidic with pH ranging around 6.5 to 7.0 decreasing to between 5.5 and 6.5 upon continued wound care.^31,32^ Accordingly, the proposed, non-antibiotic-based nanozyme treatment for infected wounds and prevention of sepsis fits in the clinical phase of infected wound treatment following debridement. To further demonstrate the dispersal properties of ZnO_2_@CeMOF/Br nanocatalysts in the absence of macrophages and their modulation, an ex vivo pilot study was carried out on supra-gingival, human orthodontic oral biofilms (Figure S10A for experimental setup). Orthodontic biofilms are highly multispecies, composed of a combination of well over 500 Gram-positive and Gram-negative commensal and opportunistic pathogenic bacterial strains.^33^ Matured supra-gingival biofilms are difficult to remove from caries susceptible sites, such as around orthodontic brackets, and are ubiquitously recognized as acidic (pH between 4.5 and 5.5 ^34^) due to the presence of cariogenic *Streptococcus mutans*.^35,36^ Substantively, 2 h of exposure of biofilms to ZnO_2_@CeMOF/Br nanocatalysts yielded significant reductions of total CFU counts and increased cell wall damage with respect to a buffer control or Triclosan (Figure S10). The ratio of log­(CFU) *S. mutans* counts, a strain highly sensitive to ROS^37,38^ over total log­(CFU) counts amounted to 0.76, and 0.81 versus a buffer control or Triclosan, respectively. This indicates that our newly developed nanocatalysts have the unique ability to selectively reduce the prevalence of highly cariogenic *S. mutans* pathogen in oral biofilms based solely on the initial generation of nonradical, low-oxdative hydrogen peroxide and the catalytic hydrolysis of phosphodiester bonds, while not affecting the overall healthy microflora in the oral cavity.

Extracellular DNA plays a crucial role in the stability of biofilms and their resistance to dispersal^39,40^ and several biofilm dispersal strategies have been proposed based on DNase cleavage of extracellular matrix DNA in infectious biofilms.^41^ However, DNase is easily inactivated under the influence of pH, ionic strength, or temperature^42^ and in vivo conditions are characterized by lower concentrations of calcium and magnesium ions than applied in many in vitro studies evaluating biofilm dispersal by DNase.^43–45^ CeMOFs do not suffer from these drawbacks.^46^ Quantitative comparison of dispersant strengths is difficult and hampered by the lack of quantitative parameters of dispersal strength and a common method for measuring dispersal.^47^ However, within these limitations, the reductions in biofilm mass observed in the literature for DNase are relatively low as compared with ZnO_2_@CeMOF/Br nanocatalysts and range between 30 and 60%.^48,49^ Dispersants like gold^50^ and iron oxide^51^ nanoparticles, graphene oxide^52,53^ and MOFs,^16^ or other metal compound nanoparticles^15,54^ operate through production of ROS, which is less specific to a particular type of biofilms, with reported biomass reduction up to 80% (see Table 1).

**Table 1:** Dispersal Strength and TNF-α and IL-6 Secretion by Macrophages in Culture Medium, Expressed Relative to the PBS Control Employed in the Respective Works^a^

		PBS ratio		
Nano- or molecular antimicrobial	Dispersal relative to PBS (%)	TNF-α	IL-6	Ref
PBS control	0	1	1	
LPS positive control	na^a^	3.0	3.0	This study
		5.0	3.0	^54^
		2.0	3.0	^62^
ZnO_2_@CeMOF/Br nanocatalysts	90	3.0	2.0	This study
Hydrogen peroxide	10–40	na	na	^63^,^64^
DNase	50	na	na	^48^,^49^
Hypobromous acid	na	3	3	This study
Gold nanoparticles	30–60	na	na	^50^
Iron oxide nanoparticles	20–80	na	na	^51^
Graphene oxide	60–80	na	na	^52^,^53^
Pt-NP/UiO-66 MOFs	90	na	4.0	^16^
CuFe_5_O_8_	60	2.0	2.0	^15^
Cu_2_MoS_4_	80	2.0	2.0	^54^
Polymeric aminoethylpiperazine nanoparticles	na	2.0	9.0	^56^
Membrane-enveloped MoS_2_ nanodots	na	17.0	na	^57^

a The table only includes data that were obtained in the absence of antibiotic assistance.

b Not applicable.

In the absence of encapsulated ZnO_2_ nanoparticles and bromide-ion loading of CeMOFs, responsible for immune modulation, up to 80% of the diabetic mice died as a result of septic symptoms arising from an infected wound. Also other dispersants without immune-modulating properties applied in the absence of antibiotic assistance demonstrated sizable death in murine studies. In an infected wound model involving *Pseudomonas aeruginosa* biofilms, the application of glycoside hydrolases to disperse the biofilm resulted in 80% mortality due to sepsis in mice.^55^ In a murine pneumonia model, 67% mice died even when treated with DNase combined with an antibiotic assist (ciprofloxacin).^49^ This attests to the fact that dispersal treatment of infectious biofilms in the absence of an antibiotic assist must be accompanied by extremely careful immune modulation in order to prevent death due to sepsis, particularly in the case of internal pathogenic biofilms. Comparing the ratios of TNF-α and IL-6 secretion by macrophages, the immune modulation achieved by our ZnO_2_@CeMOF/Br nanocatalysts is elevated (see also Table 1) yet not overly high,^56,57^ which could yield adverse effects such as rheumatoid arthritis,^58^ atherosclerosis,^59^ autoimmune diseases,^60^ or metabolic disorders.^61^

Our comparative analysis in Table 1 shows that ZnO_2_@CeMOF/Br nanocatalysts rank both among the strongest dispersants and biosafe immune modulants not causing harmful side effects in mice. According to our hypotheses (see Figure 1), ZnO_2_@CeMOF/Br nanocatalysts initially generate nonradical hydrogen peroxide for biofilm dispersal and hypobromous acid for immune modulation. Nonradical, low oxidative hydrogen peroxide, however, only cleaves extracellular matix DNA by hydrolysis of phosphodiester bonds after conversion to ROS and reportedly yields a far lower biofilm dispersal than ZnO_2_@CeMOF/Br nanocatalysts (see Table 1). This indicates that the nonradical hydrogen peroxide is not the sole responsible factor for the high dispersal observed. Consumption of ZnO_2_ nanoparticles for the generation of nonradical hydrogen peroxide exposes an intact, positively charged Ce node in CeMOFs (see Figure 4) that is crucial in the catalytic hydrolysis of phosphodiester bonds according to the catalytic reaction proposed in Figure 8A. A similar catalytic hydrolysis cycle as presented in Figure 8A has recently been proposed for CeMOFs similarly synthesized as in this work but without any further loading for the catalytic hydrolysis of phosphate ester bonds in biological phosphates and toxic phosphorus pollutants.^46^ However, in the proposed catalytic reaction by Yuan et al.,^46^ Ce^4+^ directly binds with a phosphorylic, doubly bound oxygen to weaken the phosphodiester bond and allow nucleophilic hydrolysis, without describing electron shuttle within the Ce^3+^/Ce^4+^ redox couple. In our proposed catalytic reactions, depending on the progression of the catalytic hydrolysis cycle proposed in Figure 8A, the Ce^3+/^Ce^4+^ redox couple in the Ce node will present itself as Ce^3+^ or Ce^4+^ and facilitate catalytic immune modulation by hypobromous acid (Figure 8B) that decomposes into bromide ions (and H_2_O) after having performed its modulatory function.^65^ Accordingly, both catalytic reaction cycles proposed in Figure 8 occur in the presence of Ce^3+^ or Ce^4+^ and hypobromous acid or bromide ions without being synchronized and can each be in different stages of progression.

**Figure 8:**
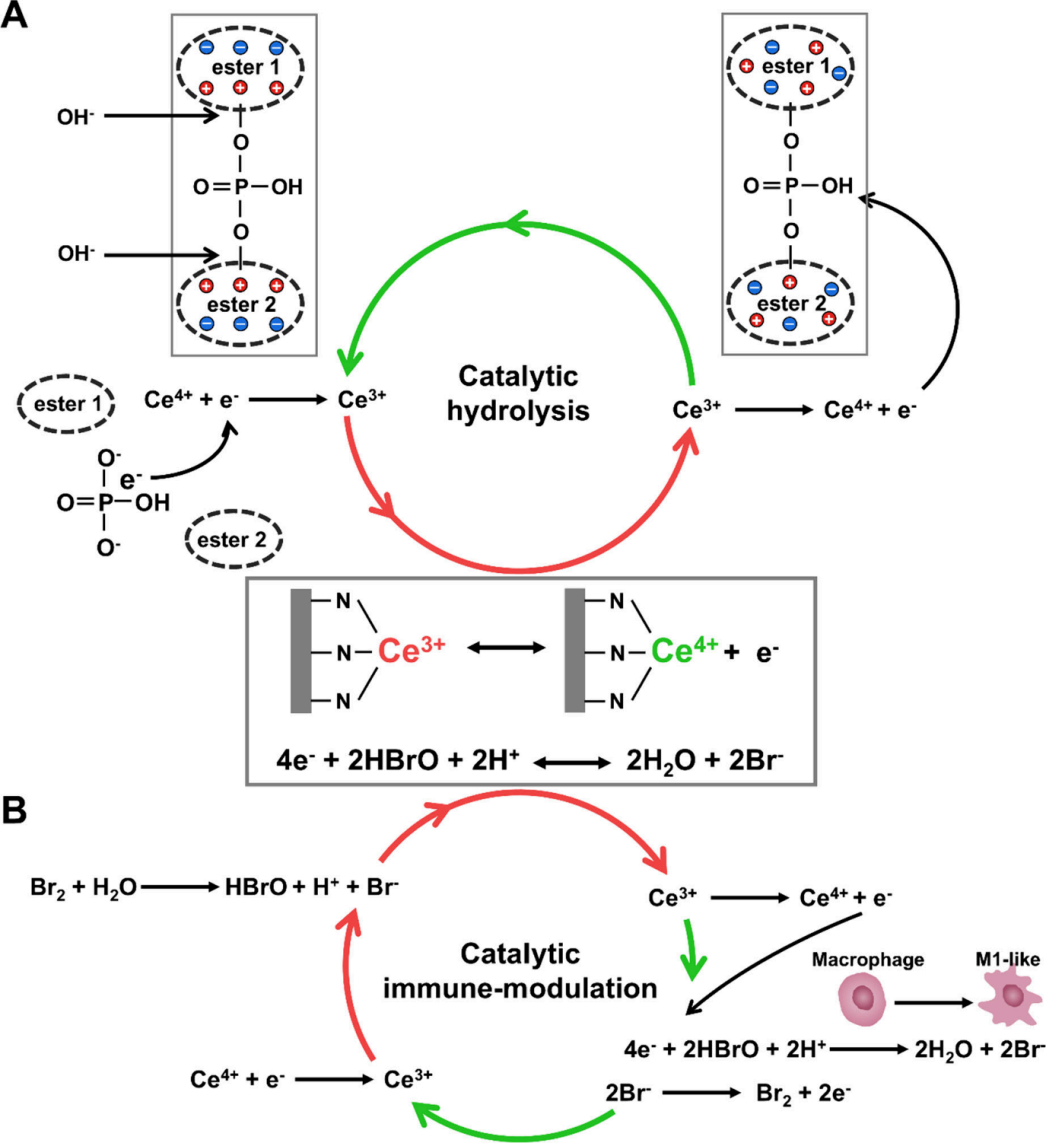
Different stages in the two catalytic reaction cycles induced by core–shell ZnO_2_@CeMOF/Br nanocatalysts in an acidic environment following initial generation of nonradical hydrogen peroxide and hypobromous acid by degradation of the ZnO_2_ nanoparticle core. Each catalytic reaction occurs in the presence of Ce^3+^/Ce^4+^ and HBrO/Br^–^, depending on the stages of progression of each reaction. (A) Catalytic mechanism responsible for the repetitive hydrolysis of phosphodiester bonds and cleavage of extracellular matrix DNA. The intact Ce node in its Ce^3+^ state is oxidized to Ce^4+^, donating an electron to the oxygen atoms in phosphodiester bonds. This adds negative charge to the phosphate group and induces polarization of the two ester groups, which makes the bond between the esters and the phosphate group attractive for interaction with nucleophiles such as OH^–^ and H_2_O, causing hydrolysis of phosphodiester bonds. After hydrolysis, the electron previously donated to the phosphate group returns to the Ce^4+^ node, converting it back into Ce^3+^. (B) Catalytic mechanism responsible for the repetitive generation of hypobromous acid and the modulation of macrophage polarization toward an M1-like phenotype. Upon performing its modulatory function, HBrO decomposes into H_2_O and Br^–^,^65^ requiring two electrons which are donated by the Ce^3+^ ions in the Ce^3+^/Ce^4+^ redox couple that is oxidized to Ce^4+^. After performing its modulatory function, the Ce^3+^/Ce^4+^ redox couple facilitates the formation of bromine and therewith returns to its Ce^3+^ state and bromine reacts with H_2_O to form hypobromous acid again.

Theoretically, catalytic reactions should have an infinitely high turnover,^66^ but experimentally this is impossible to measure due to substrate limitation, changes in experimental conditions or alterations in enzyme structure.^67,68^ Catalytic hydrolysis of BNPP by the Ce node in the CeMOF (Figure 8A) under the experimental conditions applied occurred at a relatively low turnover number and frequency of 1.2 and 1.4 × 10^–5^ s^–1^, respectively (Table S1A), but turnover was much higher at a lower CeMOF concentration, i.e., higher substrate availability (26.8 and 0.9 × 10^–1^ s^–1^, respectively; see Table S1B). Catalytic immunemodulation by the generation of transient hypobromous acid (Figure 8B) under the experimental conditions applied also occurred at a relatively low turnover number and frequency of 1.4 and 1.6 × 10^–5^ s^–1^, respectively (Table S2A), but here too turnover was much higher at a lower CeMOF concentration (14.8 and 0.5 × 10^–1^ s^–1^, respectively; see Table S2B). Despite not being synchronized, it is of interest to note that both catalytic reactions proposed occurred at the same turnover frequencies at identical nanozyme to substrate concentration ratios (Table S1 and Table S2). The high turnover under conditions with a high surplus of substrate (low CeMOF concentration) confirms the catalytic nature of reactions proposed in Figure 8.

Collectively, these two catalytic reactions proposed maintain biofilm dispersal by hydrolysis of phosphodiester bonds and cleavage of extracellular matrix DNA as well as modulation of macrophage polarization toward an M1-like phenotype by generation of hypobromous acid, as long as the experimental conditions allow. In the present study, while initiation of the catalytic reactions requires an acidic environment as in a biofilm, both catalytic reactions will come to an end after full cleavage of all matrix DNA and the exhaustion of bromide ions in the biofilm due to diffusion elsewhere in the body. These altered conditions limit the turnover which may contribute to the biosafety of these nanocatalysts observed in vivo.

## 4 Conclusion

A novel nanocatalyst, ZnO_2_@CeMOF/Br, was developed to induce the dispersal of infectious biofilms by catalytically cleaving extracellular DNA within a biofilm matrix. Dispersal was accompanied by the catalytic modulation of macrophage polarization toward a more M1-like phenotype, enhancing their ability to target and efficiently eliminate dispersed bacterial pathogens from the blood circulation. In the initial reactions controlling generation of nonradical, low oxidative hydrogen peroxide as a dispersant and hypobromous acid as an immune modulant, there was no role for the Ce^3+/^Ce^4+^ redox couple in the Ce node of the CeMOF as a catalytic center. However, the Ce^3+/^Ce^4+^ redox couple played a pivotal role in the catalytic reactions, yielding repetitive hydrolysis of phosphodiester bonds in extracellular matrix DNA and repetitive generation of transient, highly oxidative hypobromous acid. In vivo experiments demonstrated that treatment of infected wounds with ZnO_2_@CeMOF/Br nanocatalysts under low turnover conditions was biosafe without overly modulating the immune response. Infected wound treatment with ZnO_2_@CeMOF/Br nanocatalysts, even in the absence of antibiotics, effectively prevented sepsis, which is a common concern when biofilms disperse, and a large number of bacterial pathogens enter the blood circulation. Ex vivo treatments of human, orthodontic biofilms indicated the potential of ZnO_2_@CeMOF/Br nanocatalysts to eliminate acid-producing *S*. *mutans* and to restore healthy oral biofilm composition. ZnO_2_@CeMOF/Br nanocatalyst possesses high biocompatibility due to the low oxidative power of hydrogen peroxide and the transient nature of high oxidative hypobromous acid, enabling it to function without causing significant toxicity or side effects which have so far impeded clinical trials with nanozymes as a dispersant and/or modulant.^69^ This study highlights the potential of a dispersal strategy based on ZnO_2_@CeMOF/Br nanocatalysts and enable them to combat and successfully overcome bacterial infections after biofilm dispersal, without the need for additional antibiotic treatment due to the ability of ZnO_2_@CeMOF/Br nanocatalysts to modulate macrophages toward a more M1-like phenotype.

## 5 Methods

### 5.1 Materials

Poly­(vinyl pyrrolidone) (PVP, Mw = 10000), zinc acetate (Zn­(OAc)_2_, 99.99%), cerium­(III) acetylacetonate hydrate Ce­(C_5_H_7_O_2_)_3_·*x*H_2_O, 2-methylimidazole, sodium bromide (NaBr), and bis­(4-nitrophenyl)­phosphate (BNPP) were purchased from Sigma-Aldrich (St. Louis, MO, USA). Hydrogen peroxide (H_2_O_2_, 30%), ethanol (100%), and glucose were provided by Sinopharm Chemical Reagent Co., Ltd. (Shanghai, China). 3,3′,5,5′-Tetramethylbenzidine (TMB) and dimethyl sulfoxide (DMSO) were obtained from Shanghai Aladdin Biochemical Technology Co., Ltd. (Shanghai, China). Tryptone soy broth (TSB) was obtained from Hangzhou Microbial Reagent Co., Ltd. (Hangzhou, China). All chemicals were used without further purification. Ultrapure water (18.2 MΩ) was used throughout the experiments.

### 5.2 Synthesis and Characterization of Core–Shell ZnO_2_@CeMOFs and Br^–^ Loading

ZnO_2_ nanoparticles were prepared according to a previously published method.^70^ Briefly, 200 mg of PVP and 200 mg of Zn­(OAc)_2_ were dissolved in 10 mL of water and transferred to a 25 mL single neck flask. Then 1 mL of nonradical hydrogen peroxide was added and after stirring for 24 h at room temperature, the synthesized ZnO_2_ nanoparticles were collected by centrifugation at 10,000 g for 10 min at 25 °C, washed three times with ethanol and dried in vacuum at 60 °C for 12 h.

For the synthesis of CeMOFs, 5 mL of 2-methylimidazole in ethanol (41 mg/mL) and 5 mL of Ce­(C_5_H_7_O_2_)_3_·*x*H_2_O in ethanol (22 mg/mL) were mixed in a 25 mL single-neck flask and stirred for 24 h at room temperature. The CeMOFs synthesized were collected by centrifugation at 8000*g* for 10 min at 25 °C, washed three times with ethanol, and dried in vacuum at 60 °C for 12 h.

For the synthesis of ZnO_2_@CeMOFs with different ratios of ZnO_2_ nanoparticles to CeMOFs, 2 mL of a ZnO_2_ ethanol suspension (20, 10, and 5 mg/mL), 5 mL of Ce­(C_5_H_7_O_2_)_3_·xH_2_O ethanol solution (22 mg/mL) and 5 mL of 2-methylimidazole ethanol solution (41 mg/mL) were mixed in a 25 mL single-neck flask and stirred for 24 h at room temperature. The resulting ZnO_2_@CeMOFs were collected by centrifugation at 8000*g* for 10 min at 25 °C, washed three times with ethanol, and dried in vacuum at 60 °C for 12 h. These synthesis conditions yielded ZnO_2_@CeMOFs with ZnO_2_ over CeMOFs weight ratios of 2.2, 1.2, and 0.8 for ZnO_2_ suspensions with decreasing concentration, as measured using inductively coupled plasma mass spectrometry (ICP-MS, Element 2, Thermo Finnigan, Germany).

To obtain bromide-loaded CeMOFs and ZnO_2_@CeMOFs, 20 mL of CeMOFs (MOF concentration, 0.9 mg/mL) or ZnO_2_@CeMOFs (MOF concentration, 0.9 mg/mL) suspensions were mixed with 20 mL of NaBr (10 mg/mL) in a 100 mL single-neck flask and stirred for 24 h at room temperature. Then CeMOF/Br or ZnO_2_@CeMOF/Br was collected by centrifugation at 8000*g* for 10 min at 25 °C and resuspended in 5 mL of water. Initial Br^–^-ion concentration of the NaBr solution and the remaining Br^–^-ion concentration after CeMOF loading were determined using ICP-MS (see above). Experiments were conducted in triplicate.

Transmission electron microscopy (TEM), high-angle annular dark-field scanning TEM (HAADF-STEM), and energy dispersive X-ray (EDX) mapping were performed using a FEI Talos F200X microscope operated at 200 kV (FEI, Hillsboro, Oregon, USA). X-ray powder diffraction patterns were recorded on a Philips X’pert PRO MPD diffractometer applying Cu Kα radiation (wavelength 0.15406 nm). The operating voltage and current were kept at 40 kV and 40 mA, respectively. For FT-IR spectroscopy, 2 mg of ZnO_2_, CeMOFs or ZnO_2_@CeMOF nanocatalysts were mixed with 200 mg of KBr and pressed to obtain a transparent KBr pellet. FT-IR transmission spectra were collected using a HYPERION spectrometer (Bruker, Ettlingen, Germany) over the wavenumber range of 400 to 4000 cm^–1^ with a resolution of 2 cm^–1^ taking 35 scans for each spectrum. ζ potentials of the ZnO_2_ nanoparticles and different CeMOFs were measured in 10 mM potassium phosphate buffer (pH 7.4) by using a Zetasizer Nano ZS (Malvern Instruments, U.K.). To determine the BET surface area, N_2_ sorption–desorption curves were acquired at 77 K using a Micrometritics ASAP 2050 (Micrometritics, USA).

### 5.3 Biosafety Evaluation In Vitro and In Vivo

For in vitro biosafety assessment, murine NIH 3T3 (ATCC CRL-1658, Manassas, VA, USA) fibroblasts, human umbilical vein endothelial cells (HUVECs, ATCC CRL-1730), and murine macrophages J774A.1 (FH0329, Fu Heng biology, Shanghai, China) were cultured in Petri dishes filled with Dulbecco’s modified Eagle medium high-glucose (DMEM-HG, Gibco, Thermo Fisher Scientific Inc., Waltham, MA, USA) supplemented with 10% fetal bovine serum (FBS, Gibco, Thermo Fisher Scientific) and 1% penicillin/streptomycin (HyClone, Thermo Fisher Scientific). Cell cultures were maintained in a humidified 5% CO_2_ incubator at 37 °C until 70% confluency and harvested using an EDTA–trypsin solution (Solarbio, Shanghai, China), except for macrophages. Macrophages were collected using a cell scraper, followed by centrifugation at 500*g* for 10 min and resuspended in DMEM-HG. Subsequently, 8000 cells per well in a volume of 100 μL were seeded into a 96-wells plate and incubated for 24 h, and growth medium was replaced with fresh medium containing different concentrations of ZnO_2_ nanoparticles and CeMOFs with ZnO_2_@CeMOFs with different weight ratios of ZnO_2_ over CeMOFs (2.2, 1.2, and 0.8). ZnO_2_@CeMOFs nanocatalysts were synthesized only at a weight ratio of ZnO_2_ over CeMOFs equal to 1.2. After additional 24 h incubation, metabolic activity of the cells was measured using the MTT (3-(4,5-dimethylthiazol-2-yl)-2,5-diphenyltetrazolium bromide) assay (Sigma-Aldrich) following the manufacturer’s instructions, and cell viability was expressed as metabolic activity relative to the metabolic activity of medium only, set at 100%.

Hemolytic effects were evaluated *in vitro* using red blood cells from 8 week old male mice (C57BL/6J), provided by the Model Animal Research Center of Soochow University (Suzhou, China). All animal experiments were performed in accordance with the guidelines and with the approval of the Institutional Animal Care and User Committee at Soochow University (approval number 202109A0368). Anticoagulant citrate dextrose-supplemented whole blood (800 μL) was taken from the orbital venous plexus and centrifugated at 50*g* for 5 min at 4 °C to collect red blood cells. Red blood cells were then washed three times with PBS (0.01 M Na_2_HPO_4_ 0.0018 M,KH_2_PO_4_, 0.137 M NaCl, 0.0027 M KCl; pH 7.4) and suspended in 800 μL of PBS. A volume of 0.1 mL of red blood cells was mixed with 0.9 mL of different concentrations of ZnO_2_ nanoparticles and CeMOFs with ZnO_2_@CeMOFs with different weight ratios of ZnO_2_ over CeMOFs. ZnO_2_@CeMOF/Br nanocatalysts were evaluated only at a weight ratio of ZnO_2_ over CeMOFs equal to 1.2. After incubation for 3 h at 37 °C, the mixed suspension was centrifuged at 500*g* for 5 min, and the absorbance of hemoglobin in the supernatant was measured at 540 nm using a microplate reader (Synergy H1, BioTek, Winooski, VT, USA). Red blood cells suspended in PBS served as a negative control, while cells suspended in ultrapure water were used as a positive control. Percentage hemolysis was calculated according to

1 $$\mathrm{hemolysis}=\left({\mathrm{Abs}}_{\mathrm{mixed suspension}}-{\mathrm{Abs}}_{\mathrm{PBS}}\right)/\left({\mathrm{Abs}}_{\mathrm{water}}-{\mathrm{Abs}}_{\mathrm{PBS}}\right)\times 100\%$$

where Abs_mixed suspension_, Abs_PBS_, and Abs_water_ represent the absorbances of the hemoglobin in the respective suspensions at 540 nm.

### 5.4 Acid Response of Core–Shell ZnO_2_@CeMOFs with and without Br^–^ Loading

The release of Zn^2+^ and Ce^3+^ from ZnO_2_@CeMOF/Br nanocatalysts was measured using ICP-MS. ZnO_2_@CeMOF/Br nanocatalysts were added to 10 mL of potassium phosphate buffer (10 mM; pH 7.4, 6.5, or 5.5) at 25 °C (MOF concentration, 90 μg/mL). At different time points, the suspensions were centrifuged at 60000*g* for 10 min, and the concentrations of Zn^2+^ and Ce^3+^ were measured in the supernatant using ICP-MS (see above).

For the detection of nonradical hydrogen peroxide generated, ZnO_2_@CeMOFs without bromide ion loading were added to 10 mL of potassium phosphate buffers with different pH (MOF concentration 90 μg/mL; pH 7.4, 6.5, or 5.5). At different time points, suspensions were centrifuged at 60000*g* for 10 min and the concentration of hydrogen peroxide in the supernatant determined using Hydrogen Peroxide Assay Kit (Beyotime Biotechnology, Shanghai, China) (see Figure S3 for calibration curve).

Hypobromous acid generation by ZnO_2_@CeMOF/Br nanocatalysts was assessed by the oxidative bromination of phenol red into bromophenol blue. To this end, 20 μL of phenol red (20 mM) in water was mixed with 1 mL of suspended ZnO_2_@CeMOF/Br nanocatalyst (MOF concentration, 90 μg/mL) in potassium phosphate buffer (10 mM; pH 7.4, 6.5, or 5.5) at room temperature. At different time points, the mixture was centrifuged at 60000*g* for 10 min and 200 μL of the supernatant was transferred into a 96-wells plate for measuring UV–vis absorbance at 590 nm using a microplate reader (see Figure S4 for calibration curve).

### 5.5 Evaluation of Phosphodiester Bond Hydrolysis

The ability of ZnO_2_, CeMOFs, CeMOF/Br, ZnO_2_@CeMOFs, or ZnO_2_@CeMOF/Br nanocatalysts was compared by measuring the hydrolysis of bis­(4-nitrophenyl)­phosphate (BNPP), which contains a high number of phosphodiester bonds, similar to those in DNA. To this end, 10 μL of BNPP (40 mM) in water was mixed with 1 mL of suspended ZnO_2_ nanoparticles (110 μg/mL), CeMOFs (MOF concentration 90 μg/mL), CeMOF/Br (MOF concentration 90 μg/mL), ZnO_2_@CeMOFs (MOF concentration, 90 μg/mL), or ZnO_2_@CeMOF/Br (MOF concentration, 90 μg/mL) in potassium phosphate buffer (10 mM, pH 5.5) at room temperature. After 24 h, the mixture was centrifugated at 10000*g* for 10 min and 200 μL of the supernatant was transferred into 96-wells plate for determination of UV–vis absorbance at 400 nm through a microplate reader for quantitating the hydrolysis of phosphodiester bonds.

### 5.6 Bacterial Culturing, Harvesting, and Biofilm Growth

Bioluminescent *S. aureus* Xen36 (PerkinElmer, Waltham, MA) was cultured on a TSB agar plate with kanamycin (100 μg/mL) at 37 °C in ambient air. After 24 h, one colony was transferred to be inoculated with 8 mL of TSB with kanamycin (100 μg/mL) and grown for 24 h. This preculture was diluted 1:20 in 8 mL of TSB and grown for 16 h. Staphylococci were harvested by centrifugation at 4000*g* for 5 min at 4 °C and washed twice with PBS. Finally, staphylococci were resuspended in 80 mL of PBS to a final concentration of 3 × 10^8^ CFUs/mL, as quantified by plate counting in a series of separate experiments.

Staphylococcal biofilms were cultured on circular glass coverslips with a diameter of 18 mm in 12-wells plate as described before.^15^ Briefly, 1 mL of a staphylococcal suspension in PBS (3 × 10^8^ bacteria/mL) was added to the well containing the glass coverslip and left for 1 h at 37 °C under static conditions to allow bacterial sedimentation and adhesion. Then, the glass coverslip was washed with PBS and transferred into a new well containing 2 mL of TSB with kanamycin (100 μg/mL). The adhering bacteria were cultured for 24 h at 37 °C to form a biofilm. Glass coverslips with biofilm were used for further experiments.

### 5.7 Biofilm Dispersal In Vitro

Thus grown, 24 h old biofilms were exposed to fresh TSB with kanamycin (100 μg/mL) containing different concentrations of ZnO_2_@CeMOFs or ZnO_2_@CeMOF/Br nanocatalysts (MOF concentrations, 0, 45, 90, and 180 μg/mL) for another 24 h. After 24 h exposure, growth medium was removed, and biofilms were stained with SYTO9 and propidium iodide (L10316, FilmTracer LIVE/DEAD stain, Thermo Fisher Scientific) in the dark for 30 min. Then the biofilms were imaged using confocal laser scanning microscopy (CLSM, Axio Observer 7, Carl Zeiss, Oberkochen, Germany) by taking stacks of 1 μm. The volume of the biofilm was calculated by multiplying the area of the biofilm with a diameter of 18 mm (254 mm^2^) by the biofilm thickness, measured from the height of the biofilm at the *z*-axis. Furthermore, *S. aureus* Xen36 biofilms were collected from the glass surfaces by scraping using a cell scraper. The biofilm was suspended in 2 mL of PBS, serially diluted, and plated on TSB agar. After 24 h, the colony forming units (CFUs) were enumerated on the agar plates. CFU density in the biofilm remaining after dispersal was calculated by dividing the number of CFUs by the volume of the biofilm.

### 5.8 Macrophage Polarization and Phagocytosis In Vitro

Macrophage polarization was evaluated by measuring the inflammatory cytokine expression. To this end, macrophages were seeded into a 6-well plates (1 × 10^5^ macrophages per well) and incubated with DMEM-HG for 24 h. After 24 h, growth medium was replaced with fresh DMEM-HG containing ZnO_2_ nanoparticles (110 μg/mL) and CeMOF/Br, ZnO_2_@CeMOFs, ZnO_2_@CeMOF/Br nanocatalysts (MOF concentrations 90 μg/mL), lipopolysaccharides (LPS, 10 μg/mL), or hypobromous acid (100 μg/mL) and incubated for another 24 h. Subsequently, the medium was collected and centrifuged at 5000*g* for 20 min at 4 °C to precipitate cell debris and nanoparticles. The supernatant was collected and subjected to an ELISA kit (Bio-Swamp, Wuhan, China), according to the manufacturer’s protocol to quantitate the expression of TNF-α and IL-6 using a microplate reader, using calibration curves based on the absorption at 450 nm (see Figure S8 for calibration curves).

To study in vitro biofilm phagocytosis by native and immune-modulated macrophages, the ZnO_2_@CeMOFs and ZnO_2_@CeMOF/Br nanocatalysts (MOF concentrations, 0, 45, 90, and 180 μg/mL) exposed biofilms (see above) were grown for 24 h in 2 mL of DMEM-HG medium in absence (only for concentrations of 0 and 90 μg/mL) or the presence of macrophages (100000 cells/mL). Subsequently, the biofilm was analyzed using CLSM and plate counting.

### 5.9 Biosafety and Treatment of Infected Wounds in Diabetic Mice Using ZnO_2_@CeMOF/Br

In vivo biosafety and treatment of infection using ZnO_2_@CeMOF/Br nanocatalysts were examined in 8 week old, male mice (C57BL/6J), provided by the Model Animal Research Center of Soochow University (Suzhou, China). All animal experiments were performed in accordance with the guidelines and with the approval of the Institutional Animal Care and User Committee at Soochow University (approval number 202109A0368). In order to weaken the native immune system of the mice, diabetes was induced by fasting for 4 h, followed by intraperitoneal injection of streptozotocin (STZ) (120 mg/(kg of body weight) in 10 mM citrate buffer, pH 4). The following 3 weeks, mice were fed with a high-fat diet (21.8 kJ/g, 60% of energy as fat) to stimulate diabetic type 2 mice. Compared to nondiabetic models, diabetic mice tend to form thicker and more compact, persistent biofilms.^23^ The thick biofilms release a larger bacterial load into the bloodstream after dispersion by the nanocatalysts, and therewith increasing the risk of systemic infection and sepsis. This makes the diabetic model appropriate for evaluating both the biofilm-dispersal effect of our nanocatalyst and the downstream consequences of bacterial spread.

To assess the in vivo biosafety of ZnO_2_@CeMOF/Br, the backs of non-infected mice were shaved, followed by subcutaneous injection with 100 μL of ZnO_2_@CeMOF/Br nanocatalysts at a biosafe MOF concentration of 180 μg/mL. As a control, 100 μL of PBS was injected. At 72 h after the injection, mice were sacrificed, blood was collected via the orbital venous plexus and left standing for 30 min, followed by centrifugation at 500*g* for 10 min for blood cell sedimentation and for obtaining plasma for the determination of blood parameters. Also, internal organs (heart, liver, spleen, lung, and kidney) were collected for histological analysis. To this end, organs were fixed in formalin, dehydrated using a series of ethanol solutions, embedded in paraffin wax, sectioned into 4 μm slices, and stained with hematoxylin–eosin (H&E) for microscopic examination.

Next, in another group of mice, the skin of the back was lifted after shaving, and a curved scissor was used to create an open wound with 6 cm^2^ under anesthesia using intraperitoneally injected chloral hydrate (5%). Subsequently, 100 μL of staphylococcal suspension in PBS (3 × 10^8^ bacteria/mL) was dripped onto the wound to create an infection, and mice were immobilized for 30 min and individually housed for another 4 h. Mice with infected wounds were randomly divided into four groups of five mice. After 24 h (time 0 h), wounds were treated by dropping 100 μL of CeMOF/Br (MOF concentration, 90 μg/mL), 100 μL of ZnO_2_@CeMOFs (MOF concentration, 90 μg/mL), 100 μL of ZnO_2_@CeMOF/Br nanocatalysts (MOF concentration, 90 μg/mL) or 100 μL of PBS as a control on their wounds. During treatment and immediately after treatment, mice were immobilized for 30 min. The number of surviving mice was recorded every 2 h and the body weight and temperature of each mouse were recorded every 24 h, all until 72 h. At 72 h, or if they died before 72 h, all mice were sacrificed and all blood, wound tissue, and the internal organs were collected. The numbers of viable staphylococci in blood were determined using agar plating. Wound tissue of each mouse was weighed and homogenized and homogenates were plated on TSB agar after serial dilution and grown 24 h by incubating at 37 °C and the expression levels of TNF-α and IL-6 were determined using an ELISA kit.

### 5.10 Statistical Analysis

Data are expressed as means ± standard deviations (SDs). Statistical comparisons between multiple groups were conducted by a two-tailed Student’s *t* test. In vivo CFU data were analyzed using permutation statistic, because group size varied due to death of animals during the course of the study. *p* values <0.05 were considered statistically significant. Statistical analysis was performed using SPSS v.16.0 software (SPSS Inc., USA).

## Supplementary Material



## Data Availability

Data are available on request from the authors.

## Notes

Additional data such as XPS, cytotoxicity, hemolysis, different calibration curves, CLSM images of biofilms, biosafety, ex vivo biofilm treatment, and tables with turnover numbers and frequencies (PDF)
