# Supplementary material for: Core–Shell ZnO2@Cerium-Based Metal–Organic Framework with Low Turnover, Dual-Catalytic Activity for Biosafe Biofilm Dispersal and Immune Modulation
Source: ACS Appl Mater Interfaces. 2025 May 21;17(22):32111–26. doi: 10.1021/acsami.5c08974 (PMC12147083; doi:10.1021/acsami.5c08974)
Supplement: Supplementary file 1 [file am5c08974_si_001.pdf]

Supporting information

A Core-Shell ZnO_2 @Cerium-Based Metal-Organic Framework with Low Turn-Over, Dual-Catalytic Activity for Biosafe Biofilm Dispersal and Immune-Modulation

Renfei Wu[1,2], Tianjin Ge[1], Tianrong Yu[1,2], Qiaolan Shi[1,2], Rui Shi[1,2], Yijin Ren[3], Henk J. Busscher[2], Jian Liu[1]*, Henny C. van der Mei[2]*

[1]Institute of Functional Nano and Soft Materials, Jiangsu Key Laboratory for Carbon-Based Functional Materials and Devices, Soochow University, Suzhou, Jiangsu 215123, China.

[2]University of Groningen and University Medical Center Groningen, Department of Biomaterials & Biomedical Technology, Antonius Deusinglaan 1, 9713 AV Groningen, The Netherlands

[3]University of Groningen and University Medical Center of Groningen, Department of Orthodontics, Hanzeplein 1, 9700 RB, Groningen, The Netherlands

*email: jliu@suda.edu.cn; h.c.van.der.mei@umcg.nl

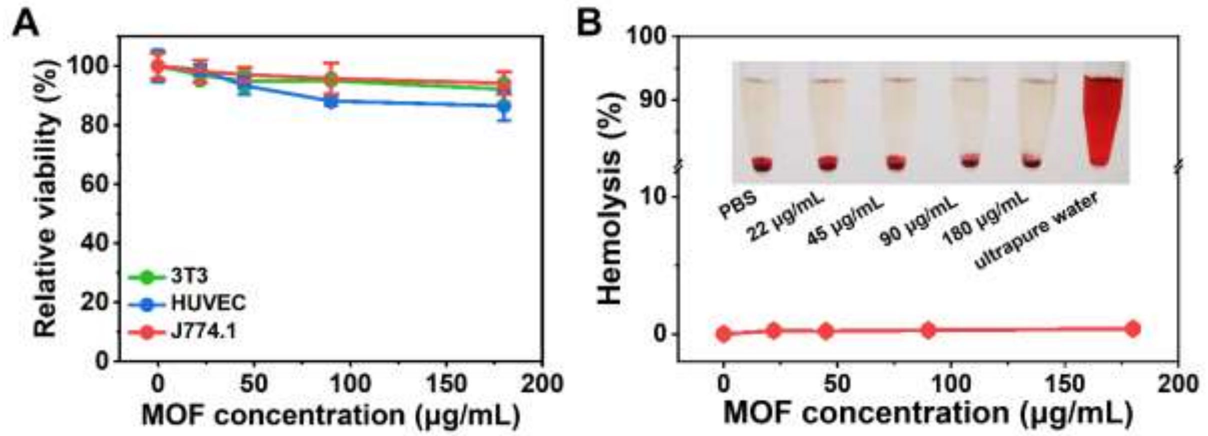

Figure S1. Cytotoxicity and hemolytic activity of bromide ion loaded ZnO_2@CeMOF/Br nanocatalysts as a function of MOF concentration up to 180 μg/mL towards NIH 3T3 fibroblasts, human umbilical vein endothelial cells (HUVEC) and J774A.1 macrophages. (**A**) Relative viability of fibroblasts, HUVECs and macrophages. Viability was expressed with respect to growth medium without MOFs, set at 100%. (**B**) Hemolysis of mouse red blood cells after 3 h exposure to nanocatalysts. Data represent means over five experiments with separately prepared cell cultures and error bars indicating standard deviations.

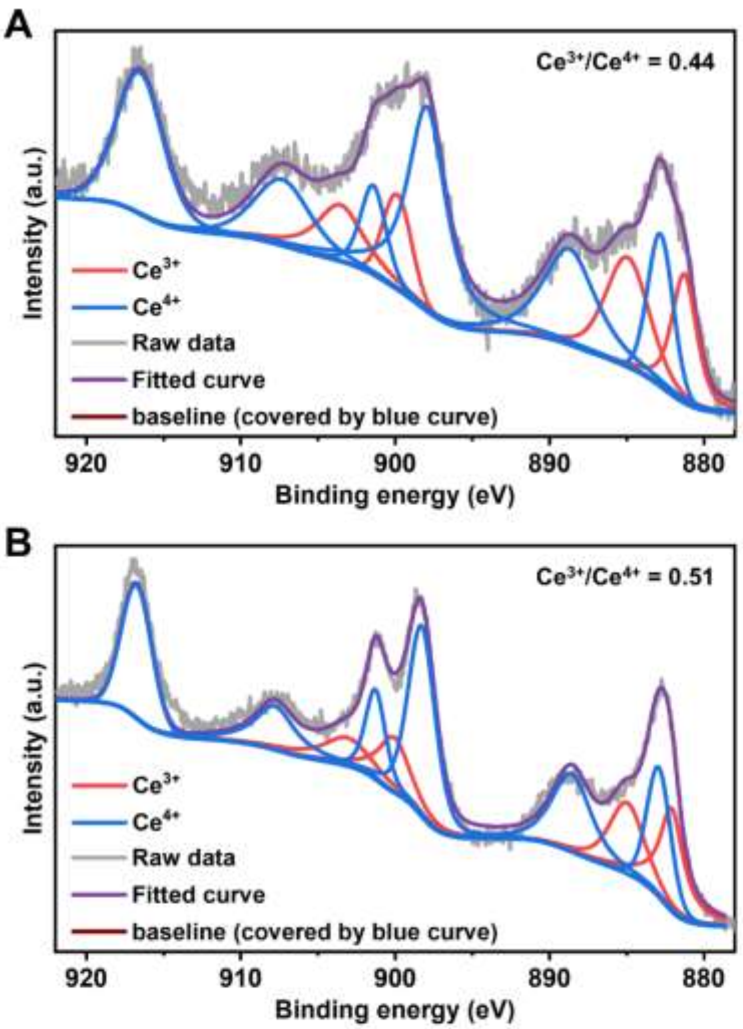

Figure S2. The oxidation state of Ce in ZnO_2@CeMOF/Br before and after catalytic conversion of H_2O_2 and Br^- ion to HBrO. The Ce_{3d} photo-electron binding energy peak was decomposed in two doublet peaks due to Ce^{3+} with a distance of 18.6 eV between peaks (red peaks at around 904 and 885 eV, 900 and 881 eV) and three doublet peaks due to Ce^{4+} (blue peaks around 917 and 898 eV, 907 and 889 eV, 902 and 883 eV). (**A**) Before catalysis, the ratio of Ce^{3+} over Ce^{4+} amounted 0.44. (**B**) After catalysis, the ratio of Ce^{3+} over Ce^{4+} amounted 0.51.

For X-ray photo-electron spectroscopy, ZnO_2@CeMOF/Br nanocatalysts were mounted onto silicon wafers. X-ray photo-electron spectroscopy was carried out using a SSI S-probe (Mountain View, CA, USA), equipped with an aluminum anode (10 kV, 22 mA) at a flood gun setting of 10 eV and a photoelectron collection angle of 55 degrees relative to the sample surface. High-resolution scans of the Ce_{3d} photo-electron binding energy peak were acquired at a pass energy of 50 eV over the binding energy range of 875 - 925 eV.

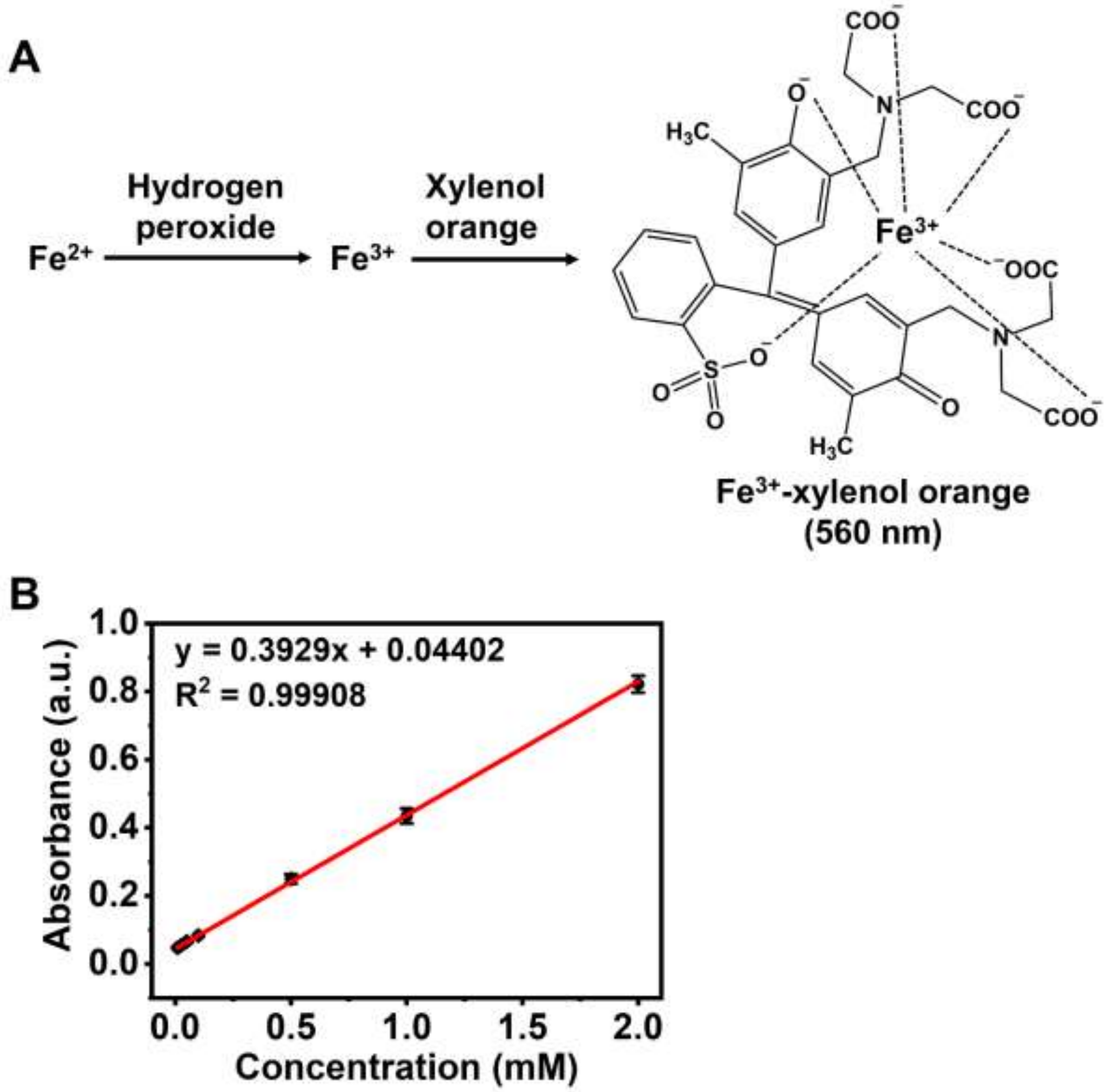

Figure S3. Calibration curve of the absorbance of non-radical hydrogen peroxide solutions (560 nm) as a function of hydrogen peroxide concentration. Absorbances were measured at a wavelength of 560 nm using a microplate reader following the manufacturer's instructions of the Hydrogen Peroxide Assay Kit (Beyotime Biotechnology, Shanghai, China). (**A**) The kit measures non-radical hydrogen peroxide concentration by oxidizing ferrous ions (Fe^{2+}) to ferric ions (Fe^{3+}) in the presence of hydrogen peroxide, which then react with xylenol orange in a specific solution to form a purple product with an absorbance at 560 nm. For the preparation of a calibration curve, 50 µL of an H_2O_2 calibration solution with concentration up to 2 mM was mixed with 100 µL Hydrogen Peroxide Assay Kit reagent at 37°C in the dark. After 30 min, UV-vis absorbance was measured. (**B**) Absorbance at 560 nm as a function of the H_2O_2 concentration. Data represent means over triplicate experiments with error bars indicating standard deviations.

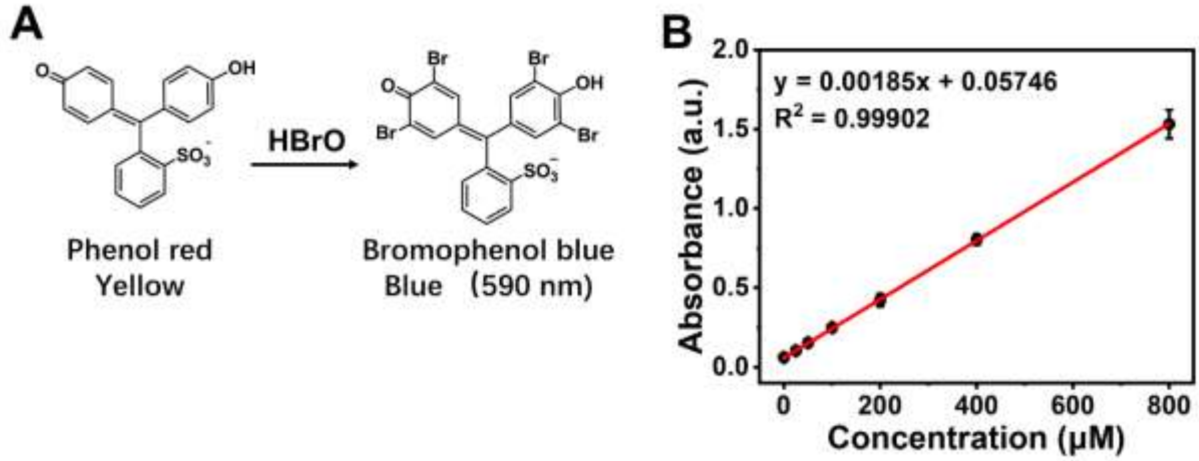

Figure S4. Calibration curve of the absorbance of bromophenol blue solution (590 nm) as a function of hypobromous acid concentration. Bromophenol blue results from the reaction of phenol red with hypobromous acid and possesses absorbance at 590 nm.[1] For the preparation of a calibration curve, 100 µL of a hypobromous acid calibration solution with concentrations up to 800 µM was mixed with 100 µL phenol red. After 30 min, UV-vis absorbance was measured. (**A**) Bromination of phenol red to bromophenol blue by hypobromous acid. (**B**) Absorbance of bromophenol blue solutions as a function of the hypobromous acid concentration. Data represent means over triplicate experiments with error bars indicating standard deviations.

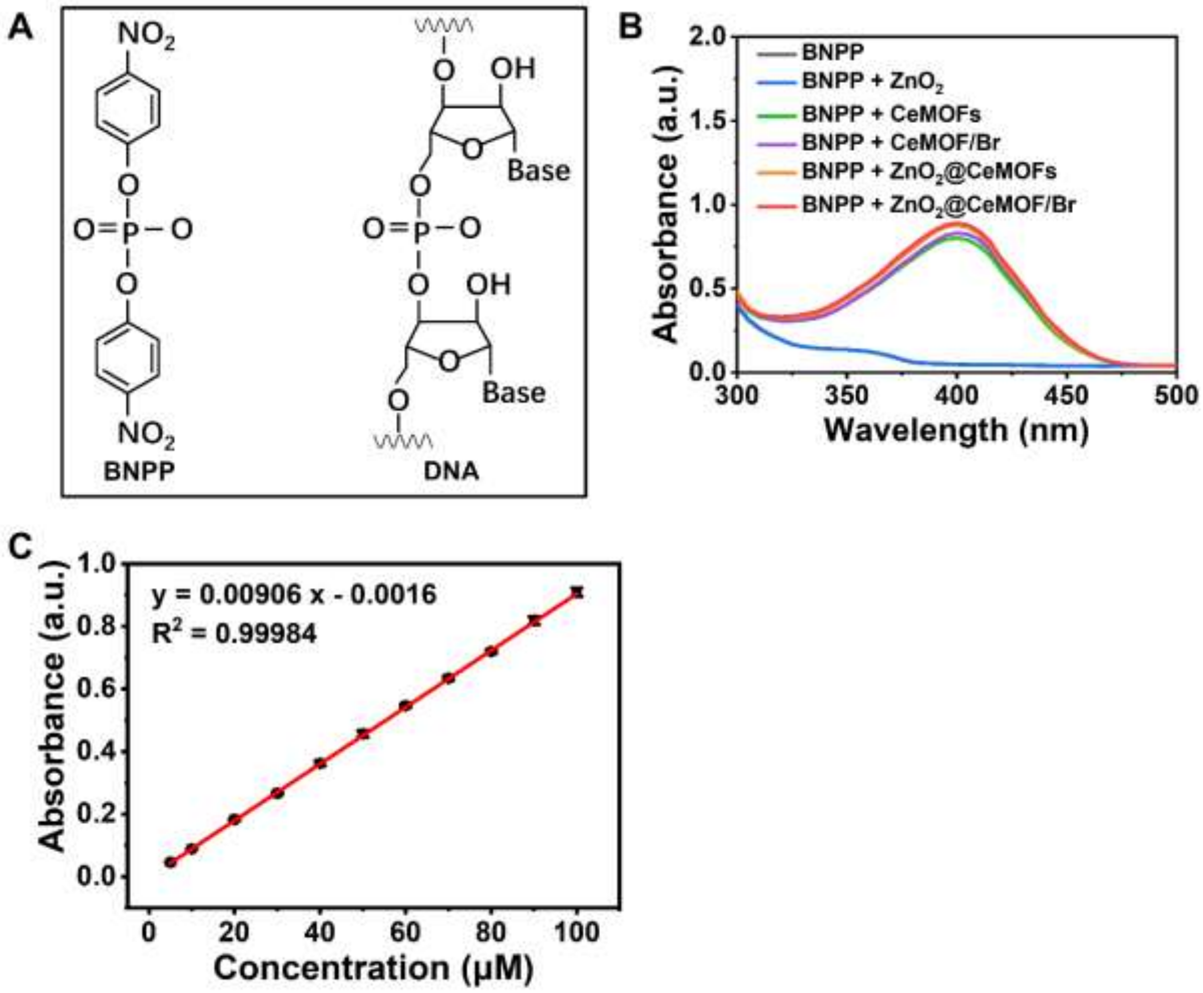

Figure S5. Hydrolysis of phosphodiester bonds by ZnO_2@CeMOF/Br nanocatalysts. (**A**) Occurrence of phosphodiester bond in bis(4-nitrophenyl)phosphate (BNPP) and DNA backbone. (**B**) UV-vis absorption spectra of nitrophenolate arising from the hydrolysis of phosphodiester bond in BNPP (0.4 mM), demonstrating hydrolysis upon 24 h exposure to ZnO_2 nanoparticles (110 µg/mL), CeMOF and ZnO_2@CeMOFs with and without Br^- ion loading (MOF concentration 90 µg/mL). (**C**) Absorbance of nitrophenolate solutions at 400 nm as a function of nitrophenolate concentration for the quantification of phosphodiester bond hydrolysis. Data represent means over triplicate experiments with error bars indicating standard deviations.

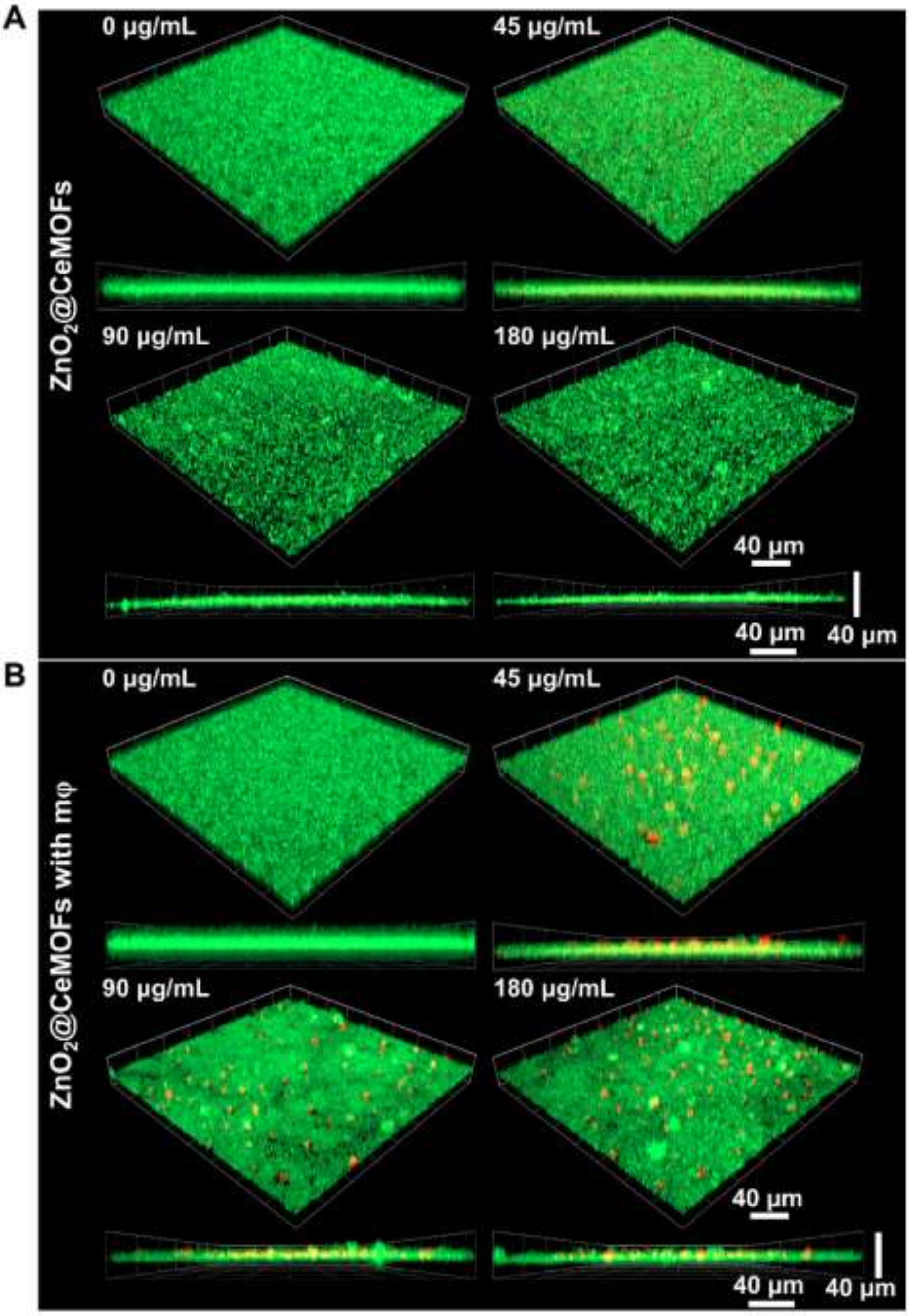

Figure S6. Dispersal of 24 h old *S. aureus* Xen36 biofilms by ZnO_2@CeMOFs *in vitro* in absence and presence of macrophages (m Φ). The biofilms were stained with SYTO9 and propidium iodide. (**A**) CLSM overlay and cross-sectional images of 24 h old biofilms exposed for 24 h to different concentrations of ZnO_2@CeMOFs. (**B**) CLSM overlay and cross-sectional images of 24 h old *S. aureus* biofilms exposed for 24 h to macrophages, following exposure to ZnO_2@CeMOFs. Note: large green or red dots are macrophages. Most macrophages display membrane damage (indicated by red-stained cells).

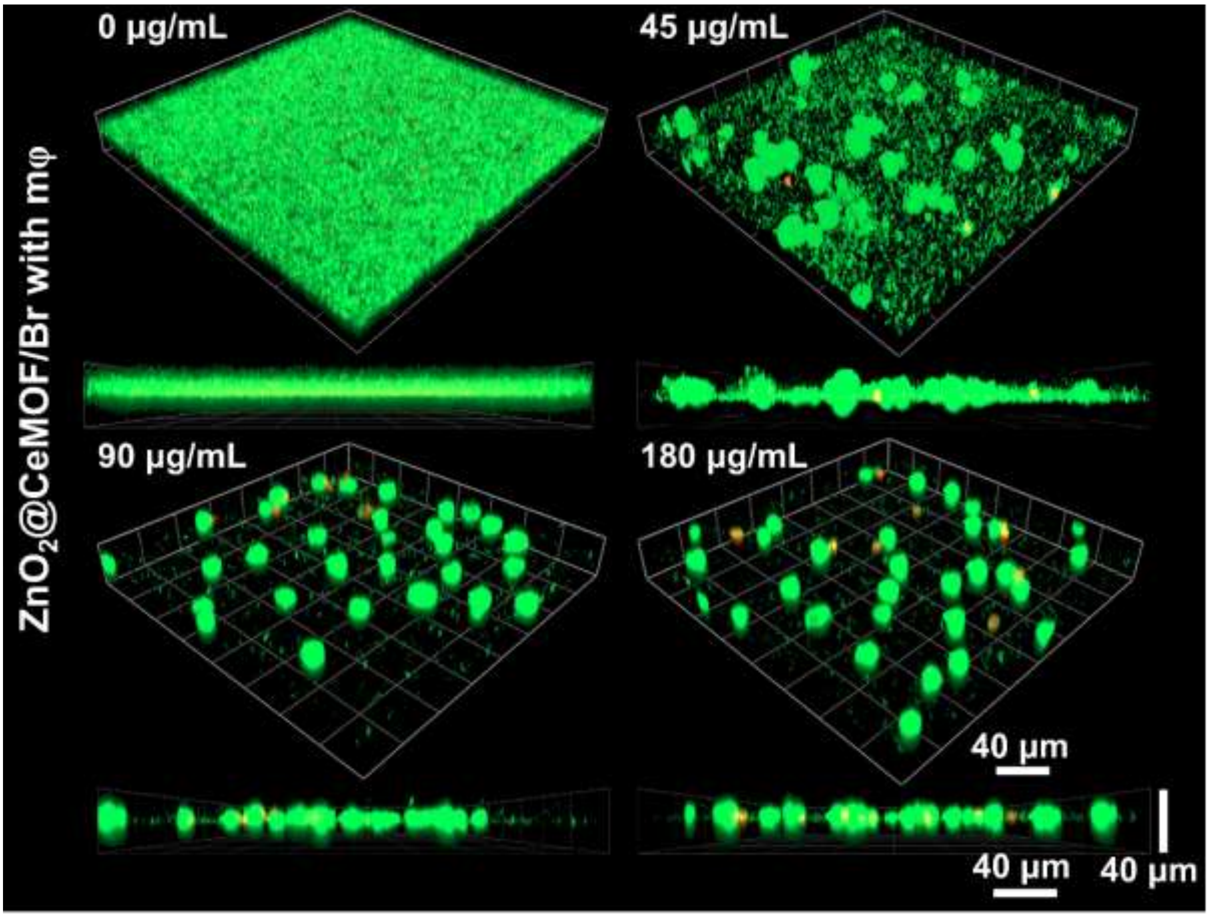

Figure S7. CLSM overlay and cross-sectional images of 24 h old *S. aureus* Xen36 biofilms exposed for 24 h to different concentrations of ZnO_2@CeMOF/Br nanocatalysts, followed by exposure to macrophages (m Φ) during 24 h. Biofilms were stained with SYTO9 and propidium iodide. Note: large green or red dots are macrophages.

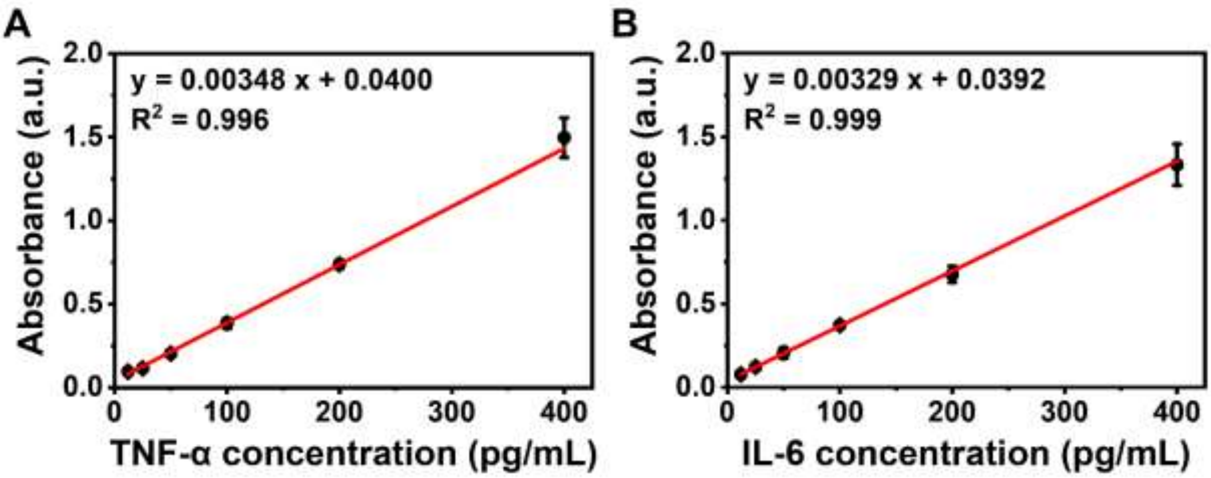

Figure S8. Calibration curves of the absorbance of cytokine solutions as a function of cytokine concentration in an enzyme-linked immunosorbent assay. Absorbances were measured at a wavelength of 450 nm using a microplate reader. (**A**) Absorbance as a function of TNF-α concentration. (**B**) Absorbance as a function of IL-6 concentration. Data represent means over triplicate experiments with error bars indicating standard deviations.

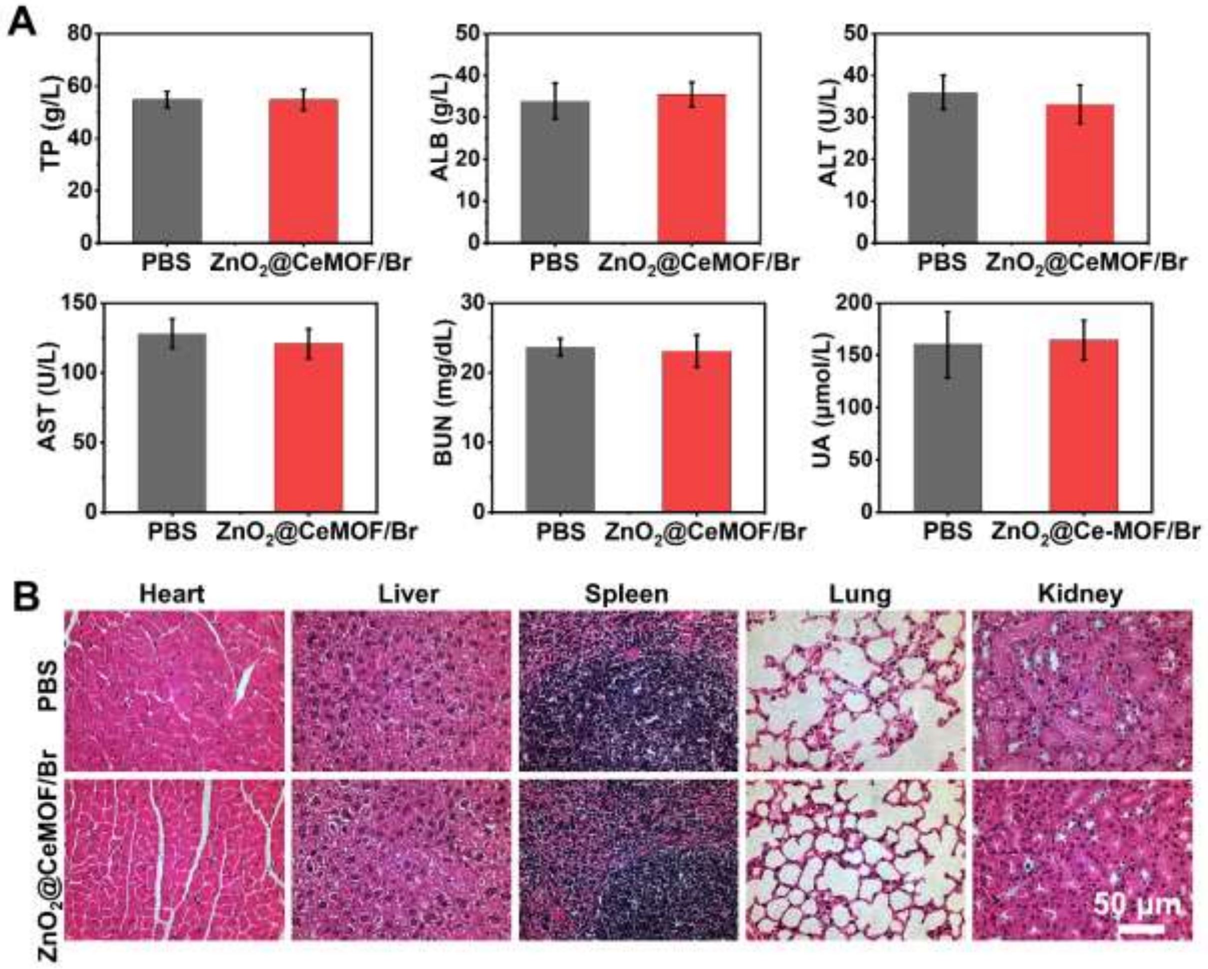

Figure S9. *In vivo* biosafety of ZnO_2@CeMOF/Br nanocatalysts. Blood biochemical and histological analyses of diabetic mice without infection. Mice were subcutaneously injected with 100 µL ZnO_2@CeMOF/Br (180 µg/mL MOF concentration) or 100 µL PBS as control. After 72 h, mice were sacrificed and blood samples collected via the eye for blood biochemical analyses. Internal organs, including the heart, liver, spleen, lung, and kidney, were collected for histological analysis after homogenization and staining with hematoxylin and eosin. (**A**) Blood parameters, including total protein (TP), albumin (ALB), alanine transaminase (ALT), aspartate transaminase (AST), blood urea nitrogen (BUN) and serum uric acid (UA). Data represent means over three mice per group and error bars indicating standard deviations. (**B**) Histological images of the internal organ tissues.

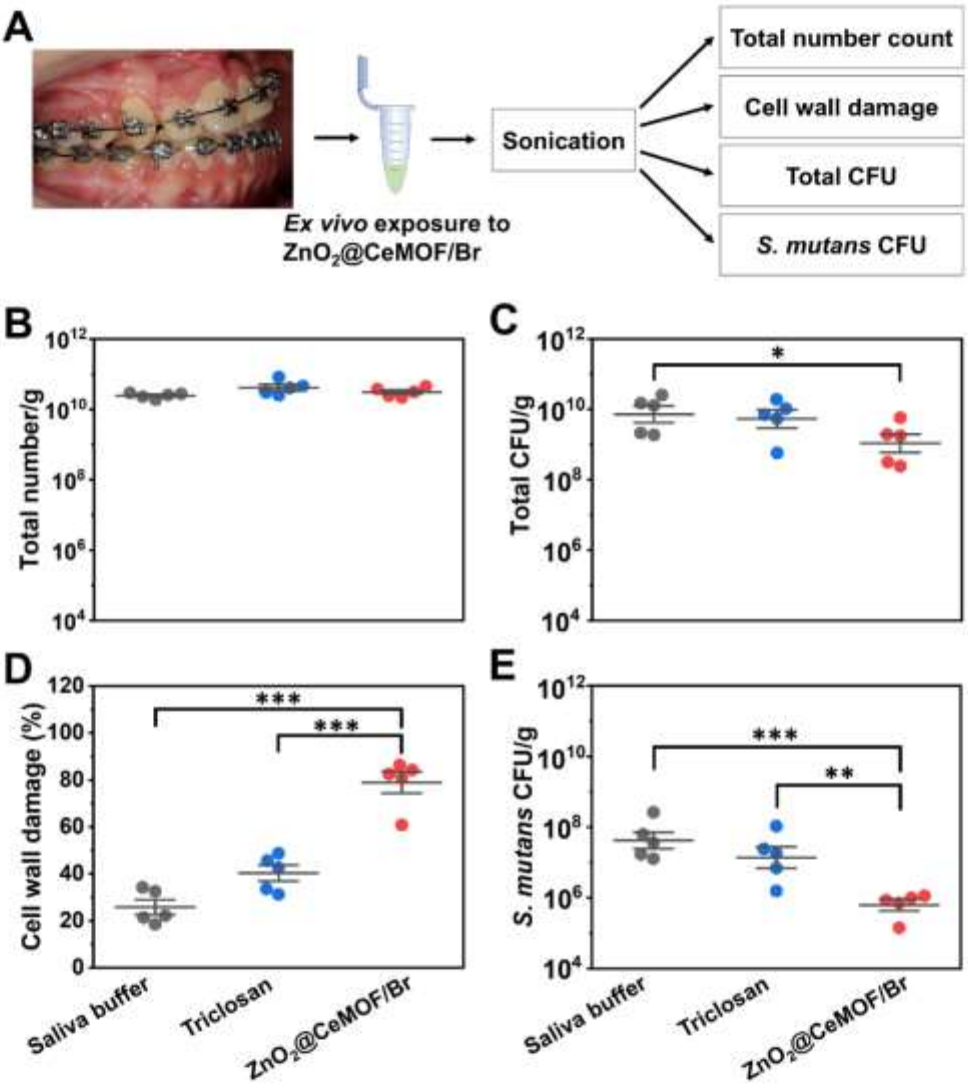

Figure S10. *Ex vivo* killing of bacteria in supra-gingival oral biofilm collected from orthodontic patients upon substantive, 2 h immersion in a ZnO_2@CeMOF/Br nanocatalyst suspension in a saliva buffer (300 µL, MOF concentration 90 µg/mL). (**A**) Overview of the experimental procedure applied. (**B**) Total counts of microscopically visible bacteria per unit weight in absence of staining in *ex vivo* orthodontic biofilms after exposure to saliva buffer, Triclosan (100 µg/mL; an oral antimicrobial[2]) or ZnO_2@CeMOF/Br nanocatalysts. (**C**) Number of colony forming units per unit weight on blood agar plates formed from *ex vivo* orthodontic biofilms after exposure, expressed per gram biofilm. (**D**) Percentage bacteria with a damaged cell wall in orthodontic biofilm samples treated *ex vivo*. Bacterial viability was quantified from fluorescence micrographs of LIVE-DEAD stained dispersed biofilms. (**E**) Number of *S. mutans* CFUs per unit weight on *S. mutans* selective tryptic soy broth agar plates from *ex vivo* orthodontic biofilms after exposure, expressed per gram biofilm. Data represent means over five orthodontic biofilm samples per group, with error bars indicating standard deviations. Statistically significant differences between pairs of data are indicated by the spanning bars (**$p < 0.01$, ***$p < 0.001$; two-tailed Student's *t*-test). This study was performed according to the guidelines of the Medical Ethics Committee of the University Medical Center Groningen, Groningen, The Netherlands (letter 17-07-2017), including the signed informed consent by the patients and according to the tenets of the Declaration of Helsinki.

Table S1. Catalytic hydrolysis of phosphodiester bonds in BNPP by CeMOFs at different BNPP substrate availability and time. (**A**) CeMOF concentration 90 µg/mL (0.3 mM Ce-nodes), BNPP concentration 0.4 mM, 24 h. (**B**) CeMOF concentration 1.5 µg/mL (0.005mM), BNPP concentration 32 mM, time 5 min).

**A** **CeMOF** 0.3 mM	*Produced in 24 h*	**Nitrophenolate formed** 0.361 mM	*Corresponding to*	**BNPP hydrolysed** 0.361 mM
**Consequently, TON =** $\frac{\mathbf{0.361\ mM}}{\mathbf{0.3\ mM}}$ **= 1.2, TOF =** $\frac{\mathbf{1.2}}{\mathbf{84600\ s}}$ **= 1.4 ×10^{-5} /s.**				
**B** **CeMOF** 0.005 mM	*Produced in 5 min*	**Nitrophenolate formed** 0.134 mM	*Corresponding to*	**BNPP hydrolysed** 0.134 mM
**Consequently, TON =** $\frac{\mathbf{0.134\ mM}}{\mathbf{0.005\ mM}}$ **= 26.8, TOF =** $\frac{\mathbf{26.8}}{\mathbf{300\ s}}$ **= 0.089 /s.**				

Turn-over numbers (TON and turn-over frequencies (TOF) were calculated according to Robert and Meunier,[3] considering only the Ce-node as the catalytic center of the nanozyme and all sites inactive in the catalytic reaction as ballast. Calculations were based on experiments carried out at pH 5.5 under the conditions of the *in vivo* experiments and conditions of increased relative substrate availability, while adding bromide ions and hydrogen peroxide since they arise as a result of the initial reactions from which catalysis starts. For the measurement of phospodiester bond hydrolysis, see Methods section in the main part of this article.

Table S2. Catalytic generation of HBrO by CeMOFs at different phenol red substrate availability and time. (**A**) CeMOF concentration 90 µg/mL (0.3 mM Ce-nodes), phenol red concentration 0.4 mM, 24 h in presence of 0.5 mM Br^{-} and 1 mM H_2O_2. (**B**) CeMOF concentration 3 µg/mL (0.01 mM Ce-nodes), phenol red concentration 1 mM, time 5 min) in presence of 160 mM Br^{-} ion and 160 mM H_2O_2.

**A** **CeMOF** 0.3 mM	*Produced in 24 h*	**Bromophenol blue formed** 0.107 mM	*Corresponding to*	**HBrO generated** 0.428 mM
**Consequently, TON =** $\frac{\mathbf{0.428\ mM}}{\mathbf{0.3\ mM}}$ **= 1.4, TOF =** $\frac{\mathbf{1.4}}{\mathbf{86400\ s}}$ **= 1.6 ×10^{-5} /s.**				
**B** **CeMOF** 0.01 mM	*Produced in 5 min*	**Bromophenol blue formed** 0.037 mM	*Corresponding to*	**HBrO generated** 0.148 mM
**Consequently, TON =** $\frac{\mathbf{0.148\ mM}}{\mathbf{0.01\ mM}}$ **= 14.8, TOF =** $\frac{\mathbf{0.148}}{\mathbf{300\ s}}$ **= 0.049 /s.**				

Turn-over numbers (TON and turn-over frequencies (TOF) were calculated according to Robert and Meunier,[3] considering only the Ce-node as the catalytic center of the nanozyme and all sites inactive in the catalytic reaction as ballast. Calculations were based on experiments carried out at pH 5.5 under the conditions of the *in vivo* experiments and conditions of increased relative substrate availability, while adding bromide ions and hydrogen peroxide since they arise as a result of the initial reactions from which catalysis starts. For the measurement of hypobromous acid, see Methods section in the main file.

References

1. Colpas, G. J.; Hamstra, B. J.; Kampf, J. W.; Pecoraro,V. L. Functional models for vanadium haloperoxidase: reactivity and mechanism of halide oxidation. *J. Am. Chem. Soc.* **1996**, *118* (14), 3469-3478.
2. Bhargava, H. N.; Leonard, P. A. Triclosan: applications and safety. *Am. J. Infect. Control*, **1996**, *24* (3), 209-218.
3. Robert, A.; Meunier, B. How to define a nanozyme. *ACS Nano*, **2022**, *16* (5), 6956-6959.
